# HIF1α regulates single differentiated glioma cell dedifferentiation to stem-like cell phenotypes with high tumorigenic potential under hypoxia

**DOI:** 10.18632/oncotarget.15888

**Published:** 2017-03-03

**Authors:** Pan Wang, Chuan Lan, Shuanglong Xiong, Xiuwen Zhao, You’an Shan, Rong Hu, Wenwu Wan, Shuangjiang Yu, Bin Liao, Guangzhi Li, Junwei Wang, Dewei Zou, Bing Chen, Hua Feng, Nan Wu

**Affiliations:** ^1^ Department of Neurosurgery, Southwest Hospital, Third Military Medical University, Chongqing 400038, China; ^2^ Department of Oncology, Cancer Hospital, Chongqing 400030, China; ^3^ Department of Neurosurgery, Xinqiao Hospital, Third Military Medical University, Chongqing 400038, China

**Keywords:** glioblastoma multiforme, hypoxia, dedifferentiation, glioma stem cell, HIF1α

## Abstract

The standard treatment for Glioblastoma multiforme (GBM) is surgical resection and subsequent radiotherapy and chemotherapy. Surgical resection of GBM is typically restricted because of its invasive growth, which results in residual tumor cells including glioma stem cells (GSCs) and differentiated cells. Recurrence has been previously thought to occur as a result of these GSCs, and hypoxic microenvironment maintains the GSCs stemness also plays an important role. Summarizing traditional studies and we find many researchers ignored the influence of hypoxia on differentiated cells. We hypothesized that the residual differentiated cells may be dedifferentiated to GSC-like cells under hypoxia and play a crucial role in the rapid, high-frequency recurrence of GBM. Therefore, isolated CD133^–^CD15^–^NESTIN^–^ cells were prepared as single-cell culture and treated with hypoxia. More than 95% of the surviving single differentiated CD133^–^CD15^–^NESTIN^–^ cell dedifferentiated into tumorigenic CD133^+^CD15^+^NESTIN^+^ GSCs, and this process was regulated by hypoxia inducible factor-1α. Moreover, the serum also played an important role in this dedifferentiation. These findings challenge the traditional glioma cell heterogeneity model, cell division model and glioma malignancy development model. Our study also highlights the mechanism of GBM recurrence and the importance of anti-hypoxia therapy. In addition to GSCs, residual differentiated tumor cells also substantially contribute to treatment resistance and the rapid, high recurrence of GBM.

## INTRODUCTION

Glioblastoma multiforme (GBM) is the most aggressive brain tumor in adults and Standard treatment comprises surgical resection followed by radiotherapy and chemotherapy. However, these strategies have minimal impact on extending the life expectancies of GBM patients [1, 2]. To protect brain function, complete surgical resection of GBM is typically impossible because of its invasive growth. This issue may cause residual tumor cells including glioma stem cells (GSCs) and differentiated tumor cells according to cancer stem cells (CSCs) theory [2]. GBM is driven by a series of GSCs with the capacity of self-renewal [3]. However, because the GSC frequency is only 11.4% [4] to 20% [5], thus we speculate that it is unlikely this small population of cells is the only causative factor that contributes to the rapid and high recurrence of GBM. As a result, differentiated cells may also promote relapse and malignant progression in glioma.

Both GSCs and differentiated glioma cells are located in a hypoxic microenvironment [6, 7]. For GSCs hypoxia plays an important role in stemness maintenance [8], and their phenotype maintenance, enrichment and tumorigenic capacity are also regulated therein [6, 9]. Unfortunately the influence of these niches on differentiated glioma cells is unclear. In 2002, Jögi *et al* [10] demonstrated hypoxia up-regulated GSC markers such as c-Kit and Notch-1 in neuroblastoma both *in vitro* and *in vivo*. Using an *in vitro* approach, Li [9] demonstrated hypoxia induced the “dedifferentiation” of differentiated glioma cells. Based on these reports, we hypothesize glioma stem-like cells may be induced through dedifferentiation under hypoxic conditions. However, studies have traditionally used cell populations (typically hundreds of cells or more) instead of single cell and have cultivated them with stem cell medium. Thus, the exact role that residual differentiated tumor cells play in the resistance and recurrence of GBM remains unclear.

Three basic features of GSCs are neurosphere formation, stemness marker expression *in vitro* and tumorigenesis *in vivo* [11]. We performed validation assays that included all the factors. In brain regions, normoxia is close to 3% O_2_ [12], and oxygen concentration in glioma becomes more serious [7]; thus, in our study, we used 1% low oxygen to investigate the effects of hypoxia on differentiated tumor cells *in vitro*, as well as their tumorigenicity *in vivo*. Because hypoxia inducible factor-1a (HIF1α) is steadily expressed under hypoxic conditions and plays a significant role in angiopoiesis and stemness maintenance [7, 13], we also determined whether HIF1α affects the process of “dedifferentiation” under 1% hypoxia.

## RESULTS

### Hypoxia induced neurospheres formation from single differentiated glioma cell

**Figure 1:**
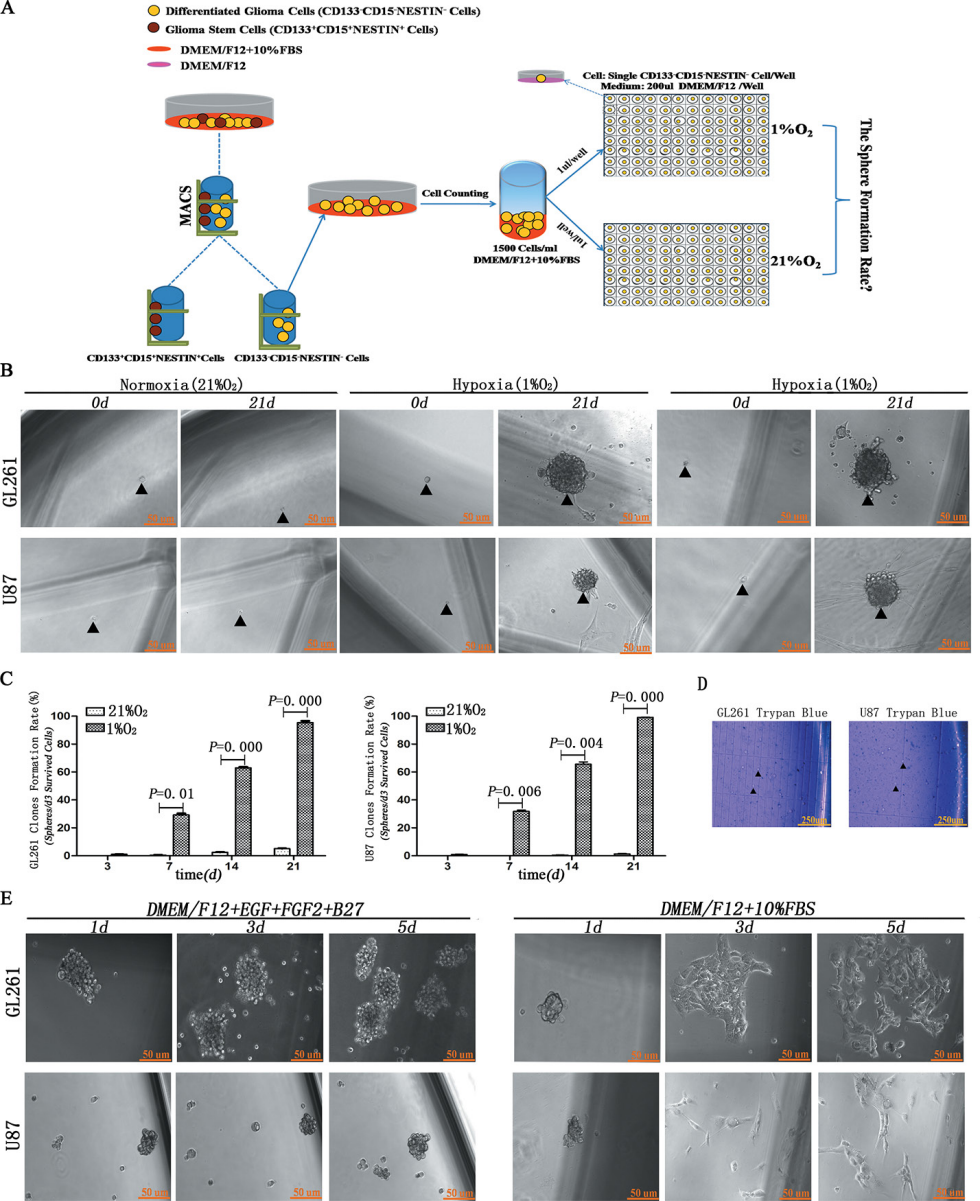
Hypoxia induced dedifferentiation of single CD133^−^CD15^−^NESTIN^−^ cell into neurospheres (**A**) Magnetic cell sorting (MACS) and hypoxia treatment strategy. Single CD133^−^CD15^−^NESTIN^−^ cell sorted through MACS was transferred to each well of 96-well plates and incubated under 1% hypoxia or 21% normoxia. (**B**) Following 21 d of incubation, the surviving single CD133^−^CD15^−^NESTIN^−^ glioma cell dedifferentiated to neurospheres under hypoxia but not normoxia. Arrow indicated seeded cell and corresponding spheroid formation. (**C**) The formation of neurospheres significantly increased under hypoxia compared with normoxia (Paired-samples *T* Test). (**D**) Trypan blue assay showed almost all the cells in neurospheres kept survival. (**E**) When hypoxia-induced neurospheres were cultured with stem cell medium, they maintained an undifferentiated sphere-like status. When cultured with 10% FBS, adherent growth and morphology were identified.

We first investigated whether hypoxia induced single differentiated CD133^−^CD15^−^NESTIN^−^ glioma cell into neurosphere. Under hypoxia more than 60% cells survived at 3 days (d). Most surviving cells (95.38% ± 5.83 from GL261 and 99.10% ± 0.78 from U87) formed neurospheres at 21 d. However, under normoxia only 5.05% ± 1.12 from GL261 and 1.31% ± 1.32 from U87 cells formed sparse, irregular and non-adherent aggregates. Most surviving cells remained a single cell and died at 21 d (Figure 1B–1C, Supplementary Figure 1 and Supplementary Table 1). Besides, the results of Trypan blue showed almost all the cells in neurospheres kept alive (Figure 1D). Asymmetric division experiments demonstrated that newly formed neurospheres grew in suspension and were maintained as spheres in stem cell medium; however, adherent growth and morphology was induced with 10% FBS administration (Figure 1E).

### Hypoxia induced an increased expression of stem cell markers

**Figure 2:**
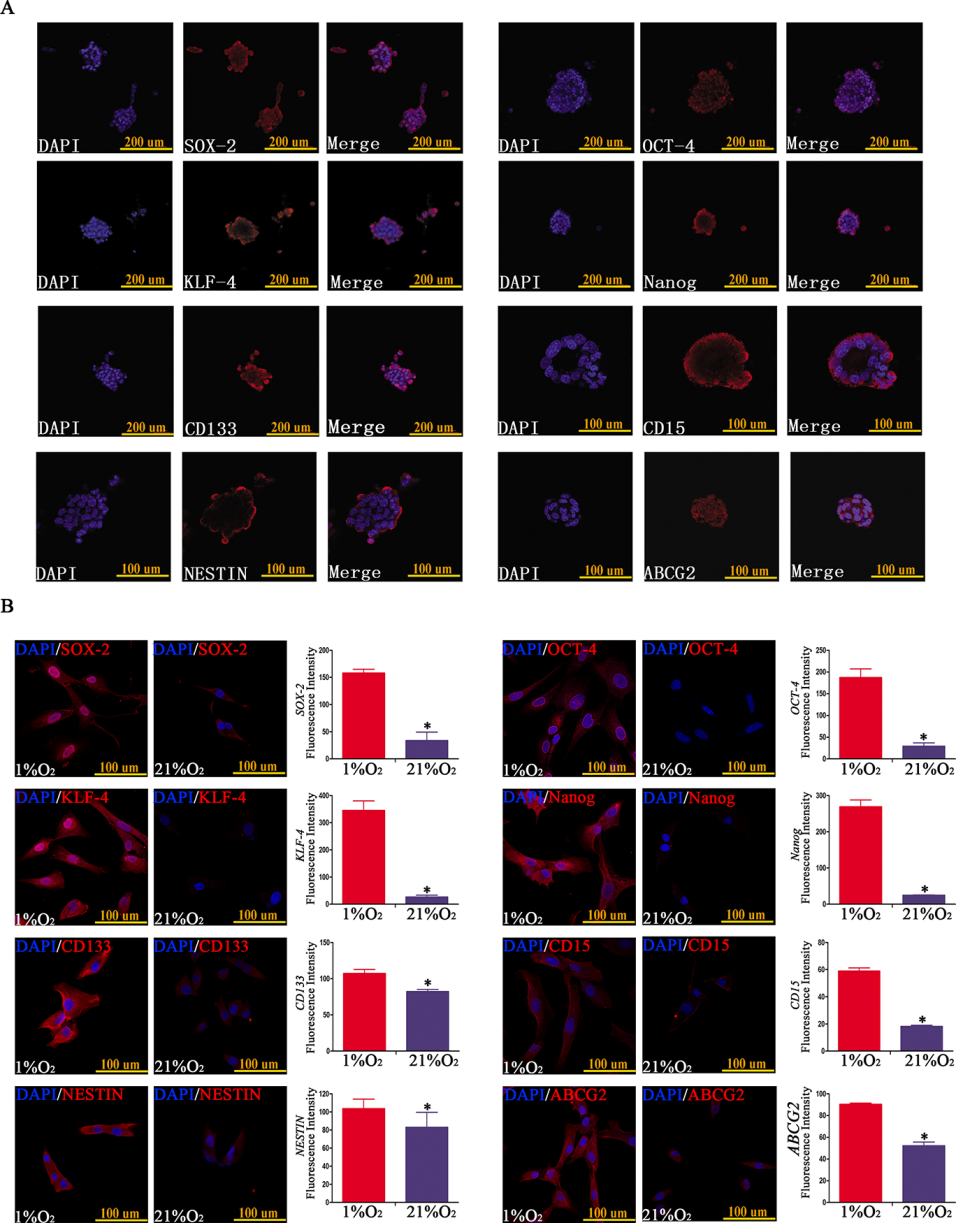
Hypoxia-induced neurospheres exhibited high expression of stem cell markers via immunofluorescence staining (**A**) Neurospheres formed by single CD133^−^CD15^−^NESTIN^−^ GL261 cell under hypoxia exhibited high expression of stem cell markers (SOX-2, OCT-4, KLF-4, Nanog, CD133, CD15, NESTIN and ABCG2). (**B**) The expression of stem cell markers of GBM CD133^−^CD15^−^NESTIN^−^ glioma cells exposed in hypoxia (1% O_2_) 48 h was higher at least 1.5-fold compared with normoxia (21% O_2_) (*P < 0.05, Paired-samples *T* Test).

SOX-2, OCT-4, KLF-4, Nanog, CD133, CD15, NESTIN and ABCG2 are frequently used as stem cell transcription factors or biomarkers of GSCs [4, 5, 14–21]. Firstly, we detected and found stem cell markers were highly expressed in neurospheres originating from single CD133^−^CD15^−^NESTIN^−^ GL261 cell after hypoxia 21 d (Figure 2A); and primary GBM CD133^−^CD15^−^NESTIN^−^ cells exposed to hypoxia 48h also increased the expression of stem cell markers compared with control in normoxia through immunofluorescence (Figure 2B). To improve the accuracy and set the detection in the same background in immunofluorescence, we did double immunofluorescent labeling of f-actin and stem cell markers for U87 CD133^−^CD15^−^NESTIN^−^ cells cultured in hypoxia and normoxia and the results showed there were no difference for the expression of f-actin between hypoxia and normoxia group; however, significant higher expression were demonstrated for SOX-2, OCT-4, KLF-4, Nanog, CD133, CD15, NESTIN and ABCG2 in the cells under hypoxia compared with the expression of stem cell markers of normoxia treated cells (Supplementary Figure 2).

**Figure 3:**
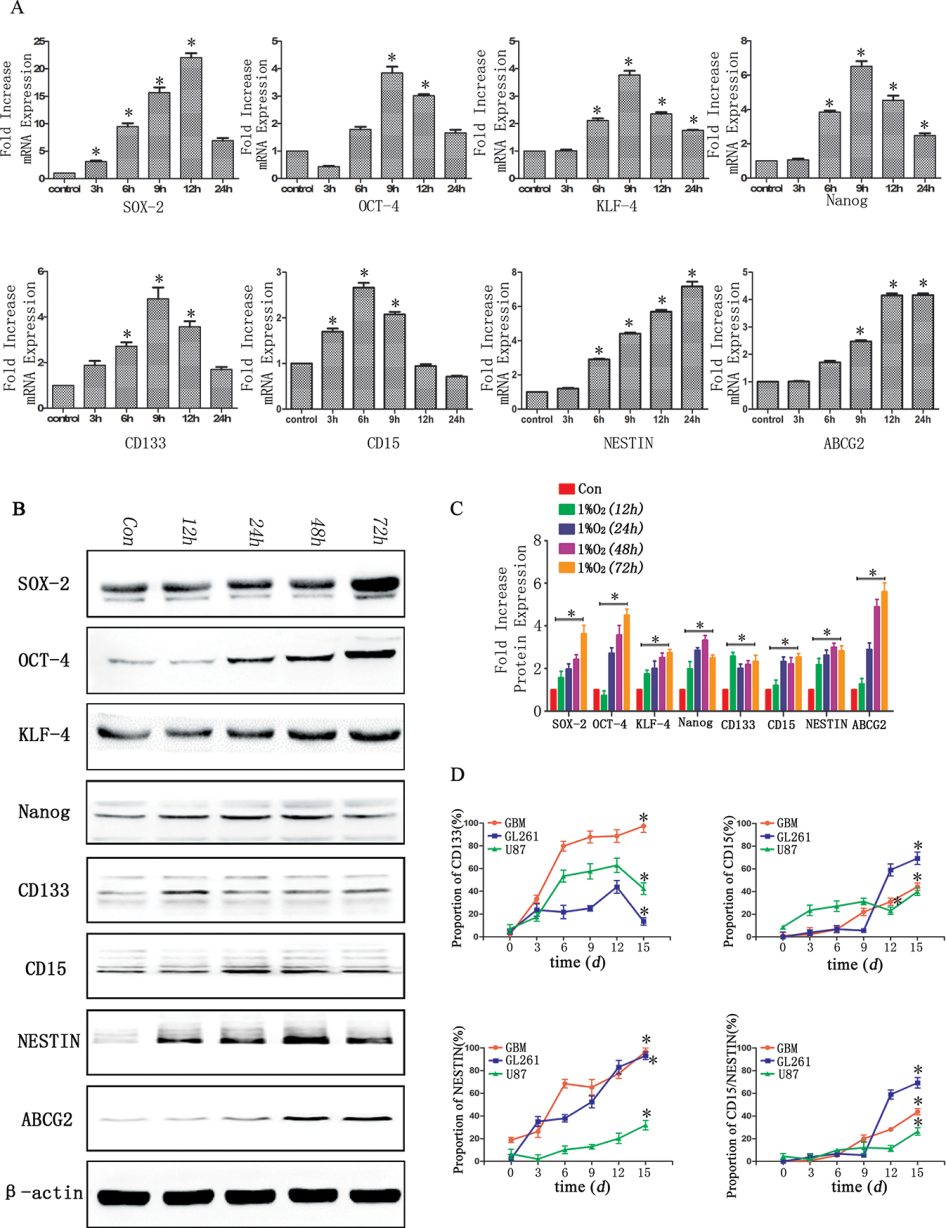
Time-dependent expression of GSC markers following hypoxia (**A**) Real-time quantitative PCR indicated time-dependent changes of stem cell markers before (con) and after hypoxia in U87 glioma cells. In general, 6 h after hypoxia, there was a significant increase of stem cell markers, which reached peak values at 9–12 h. (*P < 0.05, One-sample *T* Test). (**B**) Western blot analysis indicated a higher expression of stem cell markers after hypoxia for 12–48 h in U87 glioma cells. (**C**) Gray value analysis of Western blot in B by Quantity One indicated the expression of stem cell markers (SOX-2, OCT-4, KLF-4, Nanog, CD133, CD15, NESTIN and ABCG2) increased at least two-fold compared with control (*P < 0.05, One-sample *T* Test). (**D**) An increase expression of CD133, CD15 and NESTIN with a time-dependent manner after hypoxia (*P < 0.05, One-sample *T* Test).

Compared with normoxia controls, RT-PCR showed the expression of stem cell markers increased significantly in a time-dependent manner following hypoxia treatment for 3, 6, 9, 12 and 24 h. After 6 h of hypoxia, a significant up-regulation was identified in U87 cells, and the peak expression was detected at 9–12 h. The expression subsequently slightly decreased at hypoxia 24 h but remained statistically significant compared with control normoxia treated cells (Figure 3A). Similar results were identified with GL261 and GBM cells (data not shown).

We subsequently used Western blot to examine the expression of these markers in U87 cells exposed to hypoxia for different times (12, 24, 48 and 72 h). The protein levels of these markers sharply increased in hypoxia treatment cells and ranged from two- to six-fold higher compared with controls (Figure 3B–3C). For the GL261 and GBM cells, time-dependent increases under hypoxia were also identified for the stem cell markers (data not shown).

We cultured CD133^−^CD15^−^NESTIN^−^ glioma cells under hypoxia and detected the percentages of CD133, CD15 and NESTIN positive cells at days 0, 3, 6, 9, 12 and 15 with flow cytometry. The CD133 expression in primary glioma cells was 7.03% at day 0 (normoxia); however, its rate increased to 17.4% after hypoxia for 3 d and 62.9% after 12 d of hypoxia. The CD133 expression subsequently decreased but remained significantly increased compared with the control normoxia-treated cells. Similar results were identified in two other cell lines. The rate of CD15 positive U87 cells increased to 69.3% after 15 d of hypoxia and substantial changes were identified at hypoxia 12 d (59.055%). The CD15 expression was also significantly increased in hypoxia compared with the control in the GL261 and primary glioma cells, which increased from 1.152% to 44.226% and 8.77% to 39.9%, respectively. The NESTIN expression of U87 cells in control was only 2.252%. However, it was 35.15% at 3 d of hypoxia and then increased sharply to 93.1% at 15 d of hypoxia in a time-dependent manner. Similar results were identified in other two cell lines (Figure 3D).

### Effect of hypoxia on cell proliferation, cycle and apoptosis

**Figure 4:**
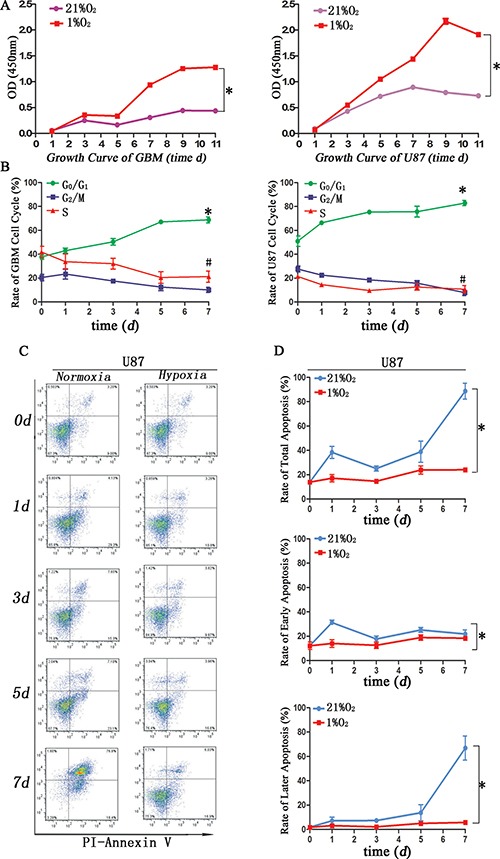
Glioma cells after hypoxia exhibited specific stem cell-like characteristics (**A**) CCK-8 assay demonstrated primary glioma and U87 cells exposure hypoxia after 5 d showed stronger proliferation (*P < 0.05, Paired-samples *T* Test). (**B**) Cell cycle analysis indicated more cells in hypoxia arrested in G_0_/G_1_ before 5 d exposure, and the rate of G_0_/G_1_ became stable after 5 d hypoxia exposure (*P < 0.05, ^#^*P <* 0.05, Paired-samples *T* Test). (**C**) Flow cytometric Annexin-V assay indicated an increase apoptosis rate in U87 cells cultured in DMEM/F12 with 1%FBS under normoxia. (**D**) Total apoptosis differed in hypoxia or normoxia for 1 d or after 5 d of exposure. Early apoptosis exhibited significant changes after normoxia for 1 d. Later apoptosis exhibited significant changes after normoxia for 5 d (*P < 0.05, Paired-samples *T* Test).

Increased proliferation [9], cell cycle arrest [22] and apoptosis resistance [15] are also stem cell-like characteristics. We used CCK-8 to evaluate cell growth under hypoxia condition, and the results showed hypoxia greatly promoted cell growth compared to that of normoxia after 5d exposure (Figure 4A). Cell cycle analysis showed hypoxia induced more cells arrested in G_0_/G_1_ but a decrease in G_2_/M+S before 5d exposure, and the rate of G_0_/G_1_ stage became stable after 5d exposure (Figure 4B). Annexin-V assay revealed lower apoptosis tendency under hypoxia exposure. Early apoptosis represented cell injury and remarkable difference came after treatment 1d, but no difference at normoxia or hypoxia 3d; later apoptosis represented cell death and obvious changes were after 5d normoxia or hypoxia exposure (Figure 4C–4D)

### Influence of hypoxia on tumorigenic through xenografts

**Figure 5:**
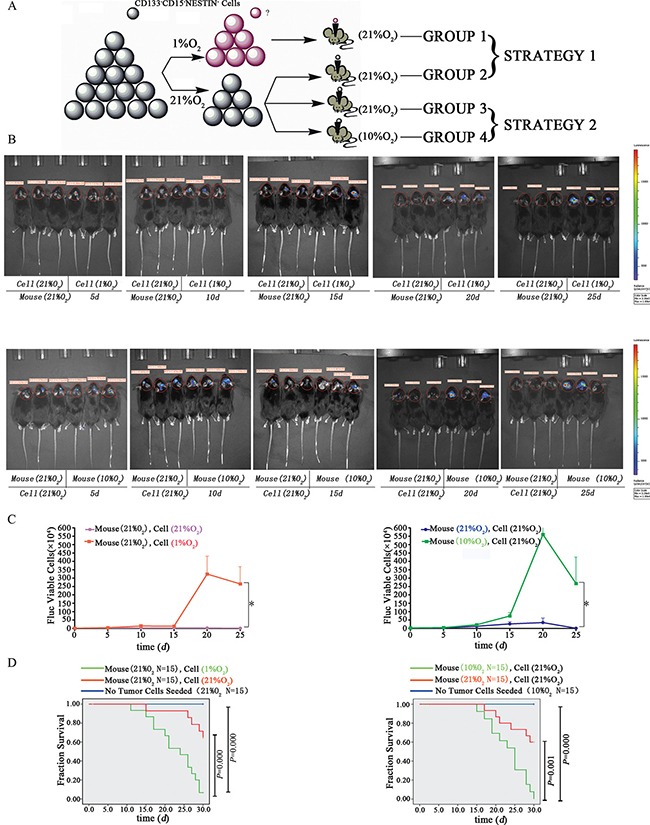
Tumorigenic potential of dedifferentiated tumor cells *in vivo* (**A**) *In vivo* tumorigenic analysis strategies. The 14 d hypoxia (1%) or normoxia (21%) exposed CD133^−^CD15^−^NESTIN^−^ Gl261-luc cells (10^4^) were injected into the brains of female C57 mice. These mice were raised under normoxia (21% O_2_). Alternatively, the mice were intracerebrally injected with normoxia-treated CD133^−^CD15^−^NESTIN^−^ Gl261-luc cells (3 × 10^4^) and subsequently raised under hypoxia (10% O_2_) or normoxia (21% O_2_). (**B**) GL261 cells following 14 d of *in vitro* hypoxia (1% O_2_) had a stronger tumorigenic ability compared with the normoxia cells. The mice with *in vivo* injections of differentiated CD133^−^CD15^−^NESTIN^−^ cells that were raised under hypoxia (10% O_2_) exhibited a stronger tumorigenic ability compared with the mice raised under normoxia. (**C**) The quantitative value of bioluminescence was significantly increased in hypoxia compared with normoxia (*P < 0.001, Paired-samples *T* Test). (**D**) Lethality analysis indicated there were significant higher death rates in hypoxia-exposed cell injection than normoxia-exposed cell injection, the same trend was found between the mice with normoxia-exposed cells raised under normoxia or hypoxia (Log-rank Test).

**Figure 6:**
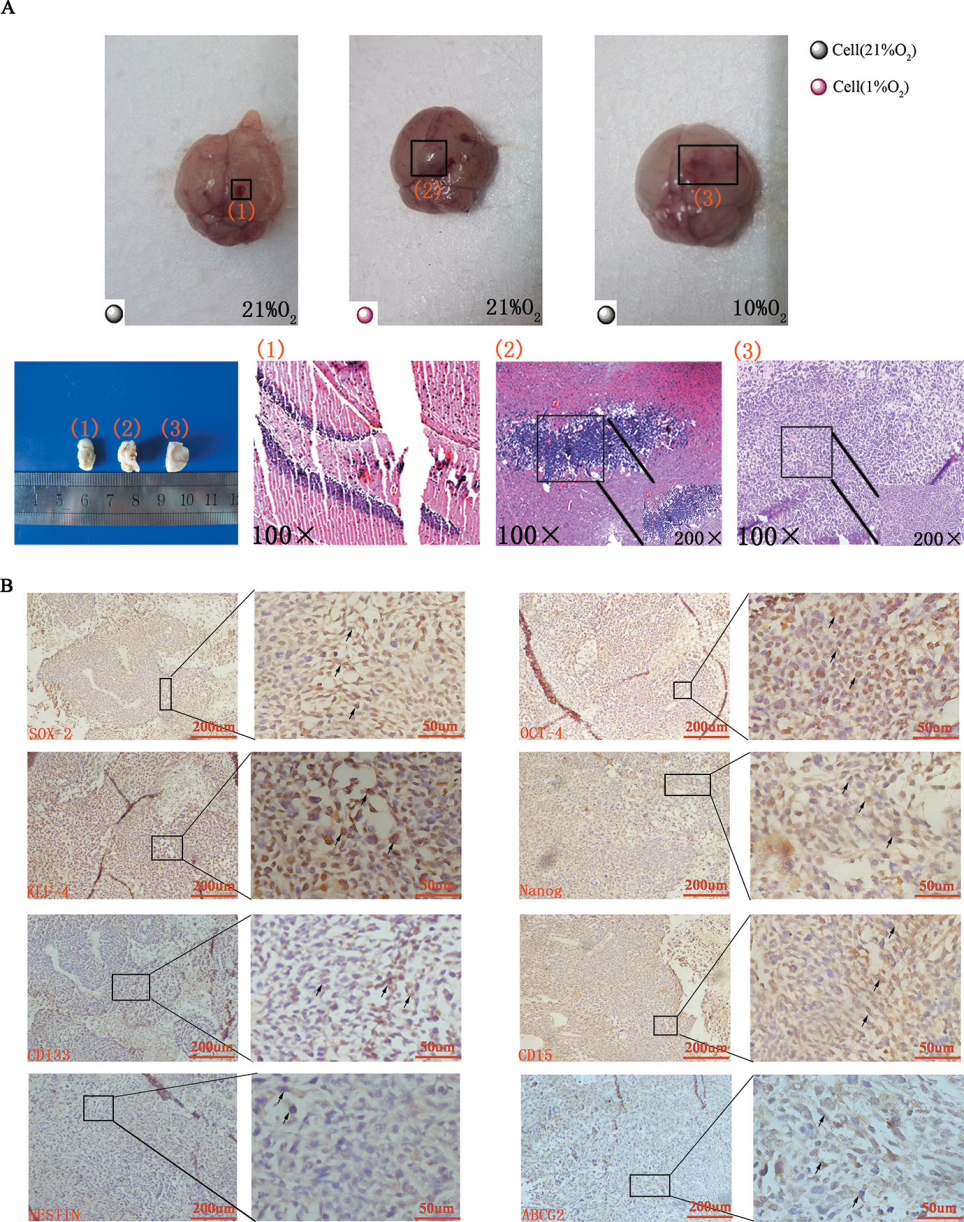
Tumor formation and immunohistochemical staining *in vivo* (**A**) Gross anatomy indicated no tumor formed when normoxia-cultured CD133^−^CD15^−^NESTIN^−^ cells were injected into the mice raised under normoxia (1 represented group 2 and 3), but tumor formed when hypoxia-cultured CD133^−^CD15^−^NESTIN^−^ cells were injected (2 represented group 1) or normoxia-cultured CD133^−^CD15^−^NESTIN^−^ cells were injected into the mice but raised under hypoxia (3 represented group 4). Similar results were demonstrated via HE staining. (**B**) Immunohistochemical staining demonstrated the increased expression of stem cell markers in tumor samples.

To investigate the tumorigenic ability of hypoxia-induced GSC-like cells *in vivo*, 14 d hypoxia (1%)-induced neurosphere–derived cells (Group 1, 10^4^) or normoxia (21%)-derived CD133^−^CD15^−^NESTIN^−^ GL261-luc cells (Group 2, 10^4^) were injected into the brains of adult female C57 mice. These mice were raised under normoxia (21% O_2_) as indicated in Figure 5A (STRATEGY 1). There were no differences on tumor volume between the two groups on day 5. After 25 d, tumors were detected in group 1 with magnitude orders more than 10^6^. There was fewer tumor formation in the second group and the order of magnitude was even less than 10^4^ (Figure 5B–5C and Supplementary Table 2). Furthermore, tumor samples and H&E staining indicated significant tumor formation in group 1 but not group 2 (Figure 6A). The mortality of the mice seeded with hypoxia cultured cells was significantly higher compared with control, and the mice without tumor cells raised in 21% O_2_ remained alive after 30 d (Figure 5D).

**Figure 7:**
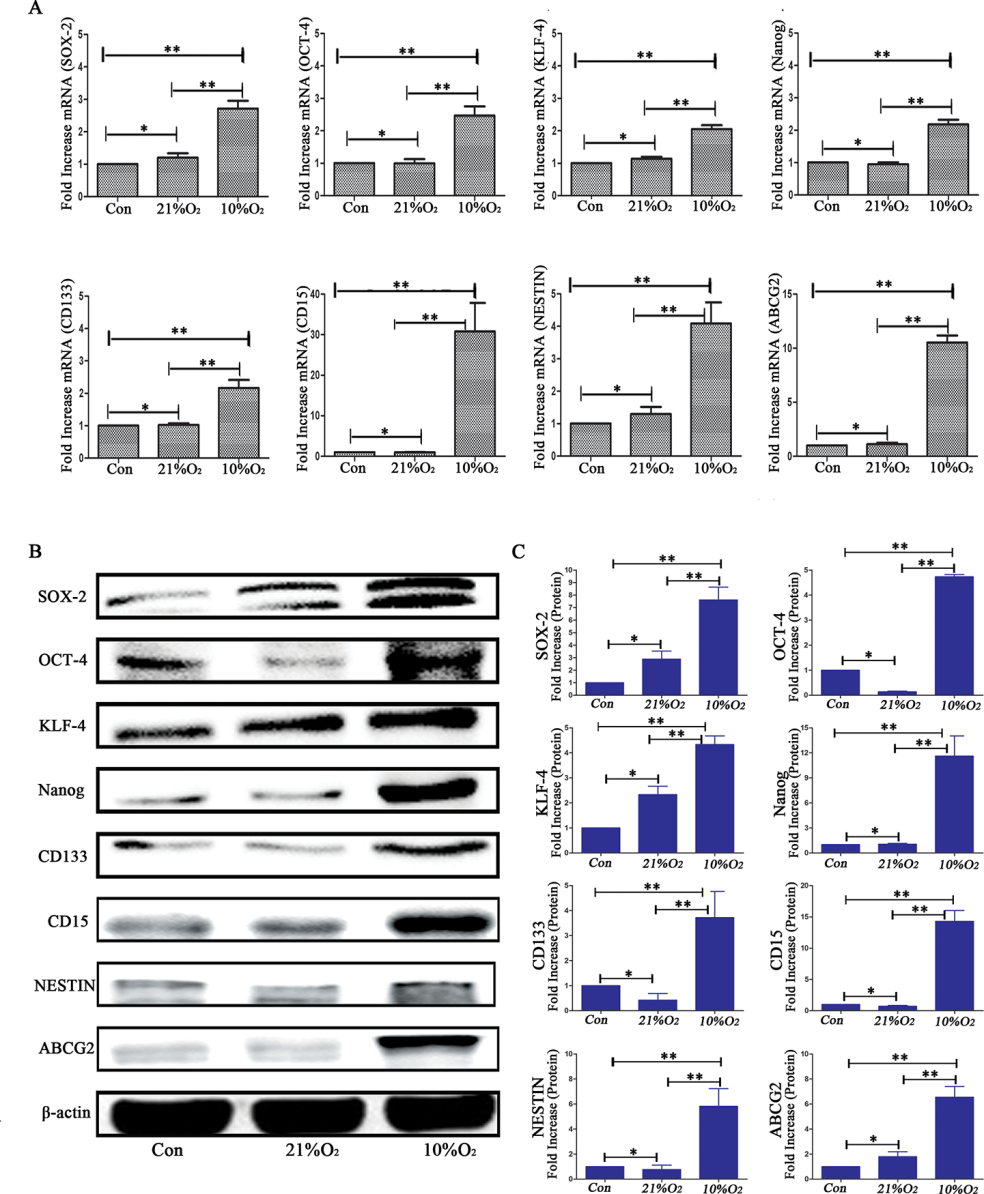
Analysis of stem cell markers in tumor sample (**A**) Real-time quantitative PCR indicated there were no significant differences on stem cell markers expression between normal control animals and normoxia mice implanted with normoxia-treated cells; however, there were significant higher expression of stem cell markers in low oxygen feeding than control or normoxia feeding (*P > 0.05, ^*^P < 0.05, One-way ANOVA). (**B**) Western blot analysis demonstrated that low oxygen feeding induced significantly increased levels of stem cell markers. (**C**) Gray value analysis of western-blot of B by Quantity One indicated the same results as real-time quantitative PCR (*P > 0.05, ^*^P < 0.05, One-way ANOVA).

In the alterative strategy (Figure 5A), the mice in group 3 (injected GL261-luc cells at 3 × 10^4^) underwent similar changes as the normoxia group in strategy 1, and group 4 was injected with differentiated CD133^−^CD15^−^NESTIN^−^ Gl261-luc cells (3 × 10^4^) but raised under 10% O_2_. At 25 d, tumors were detected in group 4 with magnitude orders greater than 10^6^. However, the order of magnitude decreased to 4,161 in group 3 (Figure 5B–5C and Supplementary Table 2). Tumor samples and H&E staining demonstrated similar results (Figure 6A). The mortality of the mice raised in hypoxia was significantly increased compared with group 3. The mice raised in 10% O_2_ without tumor cells remained alive after 30 d (Figure 5D). The expressions of stem cell markers in tumor sample were firstly determined via immunohistochemistry (Figure 6B); and RT-PCR analysis indicated there were higher expression (> 2-fold) of stem cell markers in the tumor sample collected from mice raised in 10% O_2_ (Figure 7A). Similar results were identified via western-blot (Figure 7B–7C).

### Influence of serum on neurosphere formation

**Figure 8:**
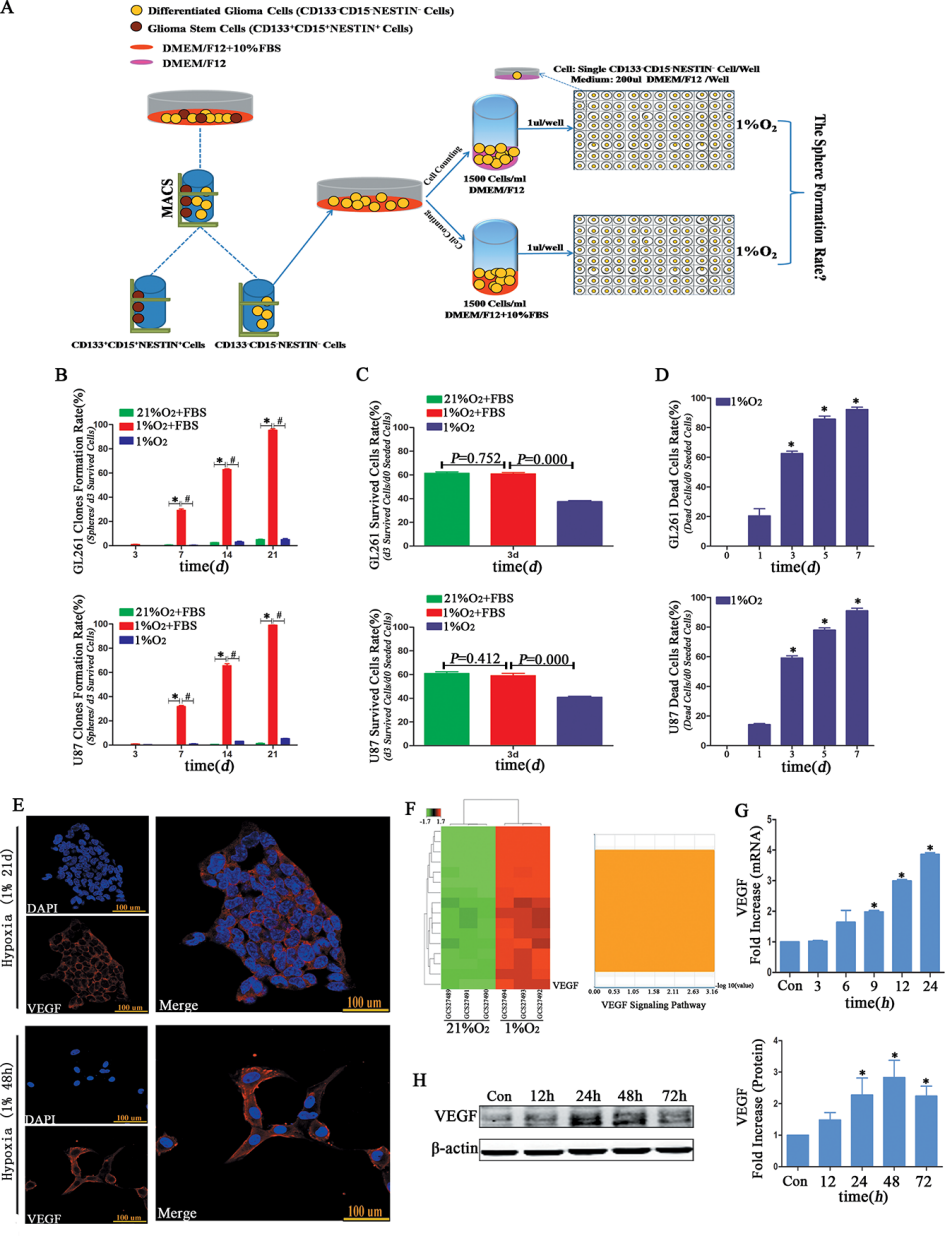
Hypoxia-induced dedifferentiation required serum in the culture medium (**A**) Different fetal bovine serum treatment strategies. One group of CD133^−^CD15^−^NESTIN^−^ cells was suspended in DMEM/F12 without FBS, and another was cultured in DMEM/F12 supplemented with 10% FBS. Single CD133^−^CD15^−^NESTIN^−^ glioma cell of each group was transferred to 96-well plates and incubated under 1% hypoxia. (**B**) The neurosphere formation rate of single CD133^−^CD15^−^NESTIN^−^ glioma cells suspended in DMEM/F12+10% FBS and cultured under 1% hypoxia was significantly higher compared with the cells suspended in DMEM/F12 without FBS or cultured in 21% normoxia (*P < 0.05, ^#^*P <* 0.05, One-way ANOVA). (**C**) The d3 cell survival rate of hypoxia-cultured glioma cells without FBS was substantially lower than the cells suspended in DMEM/F12+10%FBS, but there was no differences in the cells supplemented with FBS and cultured at 1% hypoxia or 21% normoxia (One-way ANOVA). (**D**) The glioma cell death rates without FBS sharply increased in a time-dependent manner (*P < 0.05, One-sample *T* Test). (**E**) Immunofluorescence indicated an increase expression of VEGF in neurospheres or U87 cells under 1% hypoxia 48 h. (**F**) Samples from Koh *et al* demonstrated the VEGF mRNA level sharply increased, and its rank was sixteen among all mRNA level changes. (**G**–**H**) The VEGF mRNA and protein levels significantly increased when primary GBM cells cultured under 1% hypoxia (*P < 0.05, One-sample *T* Test).

We used different strategies to investigate the influence of serum on neurosphere formation (Figure 8A). The clone formation rates (spheres/d3 viable cells) were approximately 5% from both GL261 and U87 cells suspended in medium without FBS following hypoxia treatment for 21 d (Figure 8B and Supplementary Table 3). However, the clone formation rates (spheres/d3 viable cells) of GL261 and U87 suspended with DMEM/F12+10% FBS were more than 95% (Figure 8B and Supplementary Table 3). The d3 survival rate of glioma cells without FBS was lower than that with FBS in 1% hypoxia; however, there was no difference between the normoxia and hypoxia treated cells with FBS (Figure 8C). The mortality of glioma cells suspended without FBS under hypoxia increased in a time-dependent manner and approached to 100% for both GL261 and U87 following 7 d of treatment (Figure 8D). We used immunofluorescence to examine VEGF and found its expression increased in the U87 cells after hypoxia (Figure 8E). The VEGF mRNA and protein levels of primary GBM were significantly increased in a time-dependent manner after hypoxia (Figure 8G–8H). According to the data from Koh *et al* [23], we analyzed and found VEGF mRNA level sharply increased and was ranked at 16th among the up-regulated mRNAs (Figure 8F).

### Influence of HIF1α on neurosphere formation

**Figure 9:**
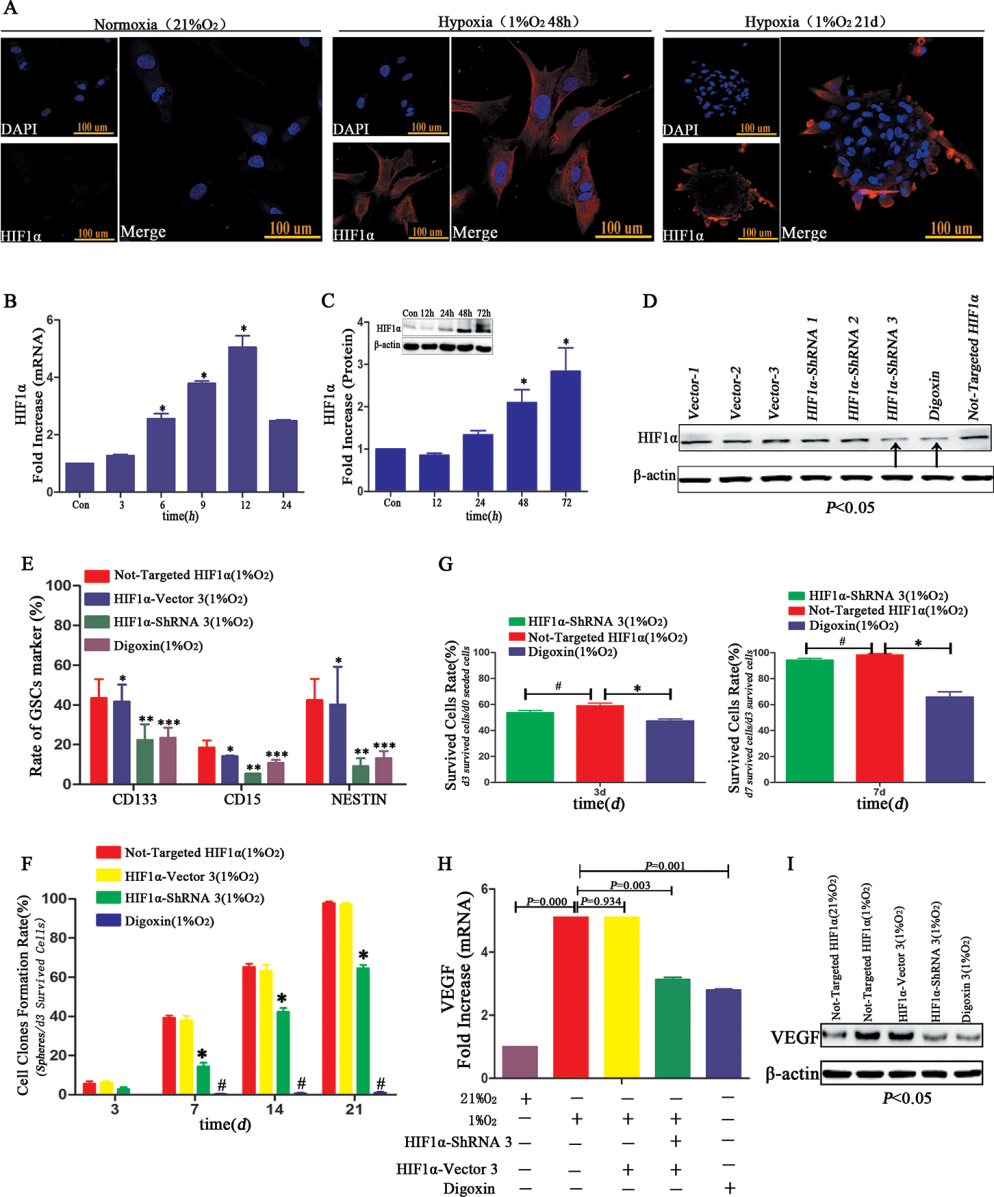
HIF1α promoted dedifferentiation under hypoxia (**A**) Immunofluorescence demonstrated GL261 neurospheres or primary GBM cultured at 1% hypoxia highly expressed HIF1α. However, the expression of HIF1α located in cell cytoplasm not nuclear as usual. (**B**) HIF1α mRNA level increased in a time-dependent manner, and the highest point was identified following hypoxia for 12 h (*P < 0.05, One-sample *T* Test). (**C**) Compared with control normoxia, HIF1α protein expressed only under 1% hypoxia conditions (*P < 0.05, One-sample *T* Test). (**D**) HIF1α-ShRNA3 and digoxin reduced HIF1α expression in U87 CD133^−^CD15^−^NESTIN^−^ cells, and vector 3 had no influence on HIF1α levels. (**E**) The rate of GSCs marker CD133, CD15 and NESTIN showed no differences between U87 CD133^−^CD15^−^NESTIN^−^ cells and HIF1α-Vector CD133^−^CD15^−^NESTIN^−^ cells after hypoxia exposure 12 d; but there existed a significant lower expression of stem cell markers in HIF1α-ShRNA CD133^−^CD15^−^NESTIN^−^ cells than control; and the digoxin group also showed a decrease of stem cell markers compared with control group (*P>0.05, ^*^P < 0.05, ^**^*P < 0.05, Paired-samples *T* Test). (**F**) The neurosphere formation rates of U87 HIF1α-ShRNA CD133^−^CD15^−^NESTIN^−^ cells and digoxin treated cells were significantly lower than control without HIF1α interference (*P < 0.05, ^#^*P <* 0.05, Paired-samples *T* Test). (**G**) There was no difference in the d3 or d7 cell survival rate (d3 surviving cells/d0 seeded cells; d7 surviving cells/d3 surviving cells) of the U87 HIF1α-ShRNA CD133^−^CD15^−^NESTIN^−^ cells compared with cells without HIF1α interference. However, the survival rate of cells treated with digoxin slightly decreased (*P < 0.05, ^#^*P* > 0.05, Paired-samples *T* Test). (**H**) VEGF mRNA expression in the U87 HIF1α-ShRNA CD133^−^CD15^−^NESTIN^−^ cells and digoxin treated cells was lower than control without HIF1α interference (Paired-samples *T* Test). (**I**) VEGF protein expression significantly decreased in the U87 HIF1α-ShRNA cells and U87 cells treated with digoxin (Paired-samples *T* Test).

High HIF1α expression was identified through immunofluorescence in hypoxia-treated GL261 neurospheres or primary GBM cells after hypoxia for 48 h, which expressed in the cell cytoplasm, not in nuclear as usual (Figure 9A). RT-PCR indicated the peak HIF1α expression of more than 5.5-fold occurred after 12 h of hypoxia (Figure 9B). The same trend was demonstrated via Western blot (Figure 9C). Next, we successfully silenced HIF1α through HIF1α-ShRNA or digoxin in U87 cells (Figure 9D). To determine the influence of HIF1α on dedifferentiation, we examined neurosphere formation rate of U87 HIF1α-ShRNA CD133^−^CD15^−^NESTIN^−^ cells under hypoxia conditions. And we found the neurosphere formation rate of the HIF1α-ShRNA- or digoxin-treated U87 cells was substantially lower than controls. The numerical value was 63.55 ± 3.59% from the single CD133^−^CD15^−^NESTIN^−^ HIF1α-ShRNA U87 cells and 1.94 ± 0.65% from digoxin treatment (Figure 9F and Supplementary Table 4). And the proportion of stem cell markers including CD133, CD15 and NESTIN decreased significantly in CD133^−^CD15^−^NESTIN^−^ HIF1α-ShRNA U87 cells after hypoxia 12d (Figure 9E). HIF1α-ShRNA had no statistically effect on cell survival rates (d3 surviving cells/d0 seeded cells; d7 surviving cells/d3 surviving cells) compared with normal HIF1α expression group; however, the cell survival rate reduced under digoxin (Figure 9G). We then detected and found the VEGF expression of HIF1α-ShRNA and digoxin treated U87 cells was lower than controls at both mRNA and protein levels (Figure 9H–9I).

### The influence of HIF1α on tumor formation

Firstly, HIF1α expression was detected in tumor sample collected from mice fed in 10% O_2_ through immunohistochemistry (Supplementary Figure 3A). Western-blot and RT-PCR were used and we found HIF1α expression of the tumor sample raised in hypoxia was about three to four times higher than control including normal brain tissues and tumor sample obtained from normoxia (21% O_2_) raised mice (Supplementary Figure 3B–3C). Then we implanted 3 × 10^4^ GL261-luc HIF1α-ShRNA cells into C57 mice brain and observed that the tumor size was much smaller than control groups with the same tumor cells seeded. The orders of magnitude all reached to 3 × 10^6^ in the control group, however the magnitude orders were less than 1 × 10^6^ in GL261 HIF1α-ShRNA cells seeded group (Supplementary Figure 3D–3E). Besides, the GL261 HIF1α-ShRNA cells seeded group showed higher survival rate (Supplementary Figure 3F).

## DISCUSSION

Hypoxic microenvironment exists in glioma and this becomes more serious for residual glioma cells after surgery because of vascular damage [24, 25]. It is widely accepted that lower oxygen is a major reason for chemo-radioresistance through stemness maintenance in glioma patients [8]. For example, 7% oxygen concentration induced higher levels of CD133 and other stem cell markers such as Sox2 and Oct4, and increased the ability of GSCs to form neurospheres compared with an atmospheric oxygen level (20%) [6]. For glioma-derived CD133^−^ cells, both Blazek [26] and Mathieu [27] demonstrated hypoxia significantly increased CD133 expression and induced neurosphere formation. However, these studies cultured hundreds of cells in stem cell medium (DMEM/F12+EGF+FGF2+B27); thus, it is not clear whether the enhanced CD133 originated from natural CD133^+^ cells or differentiated tumor cells. Moreover, the up-regulation of CD133 may be influenced by EGF and FGF2 in stem cell medium, so what's the influence of hypoxia microenvironment on differentiated glioma cells is not clear. To further validate our study, we sorted single CD133^−^CD15^−^NESTIN^−^ differentiated glioma cells and performed our experiments under 1% hypoxia without growth factors in DMEM/F12 culture medium (conditioned medium).

GSCs are capable of neurosphere formation and clone proliferation [19], which is a vital prerequisite for GSCs. We first examined the neurosphere formation rate of differentiated glioma cells under 1% hypoxia in conditioned medium. When single CD133^−^CD15^−^NESTIN^−^ cell was cultured under hypoxia for 21 days, more than 95% of surviving cells were induced to form neurospheres. Even if 20% of the single glioma cells were GSCs as reported [4, 5], the other 75% of the newly formed neurospheres identified in our studies should be dedifferentiated from differentiated non-GSCs. Moreover, these CD133^−^CD15^−^NESTIN^−^ differentiated cells were sorted through MACS three times. Therefore, the GSCs rate should be less than 20%. Another basic feature of GSCs is biomarker expression. CD133-positive cells as GSCs have been studied in many traditional studies [4, 5, 28]. However, the reliability of CD133 as a stem cell marker remains questionable because CD133^−^ cells may be tumorigenic in some cases [29, 30]. Although some researchers attributed this to CD133^+^ cell contamination as a result of missorting [31], we fully considered this issue and sorted with other GSC markers including SSEA-1/CD15 [16], NESTIN [21, 32] and ABCG2 [17], as well as several known dedifferentiation stem cell markers including SOX-2, OCT-4, KLF-4 and Nanog [18, 21]. Both induced neurospheres and cells cultured at 1% hypoxia for 48 h expressed all the stem cell markers, which indicated hypoxia induced neurospheres possessed of stem cell features. The most important biological function of GSCs is their tumor formation ability *in vivo* [3]. Singh *et al* [5] developed a xenograft assay and demonstrated that brain tumor initiating cells initiated tumors *in vivo*. We used two different strategies to identify the tumorigenic ability of glioma cells under hypoxia exposure and found glioma cells derived from hypoxia exposure or mice injected with CD133^−^CD15^−^NESTIN^−^ cells raised under hypoxia induced significant tumor formation with increased lethality and GSC markers expression. This *in vivo* study further demonstrated hypoxia dedifferentiated tumor cells into functional GSCs.

To further investigate the stem cell biology of these hypoxia induced cells, we employed asymmetric division [33], cell proliferation[9] and cycle arrest [22] as well as apoptosis analysis [15]. Beier *et al* [34] reported that GSCs displayed neurosphere-like and asymmetrical divisions. Our results showed these newly-formed neurospheres presented self-renewal and extensive proliferation in serum free medium containing EGF and FGF2 but the differentiation could be induced with serum. Additionally, proliferation analysis with CCK-8 showed hypoxia greatly promoted cell growth after 5d exposure and cell cycle analysis showed hypoxia induced more cells arrested in G_0_/G_1_ but a decrease in G_2_/M+S. Finally, Annexin-V assay revealed lower apoptosis tendency under hypoxia exposure. These results further demonstrated the induced neurospheres were stem cells, indicating single CD133^−^CD15^−^NESTIN^−^ cell was successfully dedifferentiated into CD133^+^CD15^+^NESTIN^+^ GSCs by hypoxia and these neurospheres possessed the characteristics of stem cells.

In CSC theory, researchers hypothesize CSCs divide asymmetrically, whereas differentiated cells grow symmetrically [11]. However, several recent findings have challenged the idea because they identified a dynamic interconversion between CSCs and differentiated cells, which indicated the differentiation of CSCs was not the only way in which cells grew and a reversible dedifferentiation process may occur through special signals [30, 32, 35, 36]. These findings were in accordance with our results as we found cancer stem cells can be induced through dedifferentiation under hypoxia conditions. We also challenge the traditional glioma cell heterogeneity model that includes a stochastic model as all tumor cells may develop mutations to promote tumor growth, and a hierarchical model in which only GSCs contribute to tumor growth [37]. In a new theory based on our results, we agree with the stochastic model that each cell is associated with tumor maintenance; however, differentiated glioma cells participate this through a special dedifferentiation way in some microenvironments such as hypoxia. Moreover, differentiated tumor cells are formed by GSCs in specific situations such as serum presence. This viewpoint is in accordance with two recent studies which demonstrated GSC-like cells may be induced by non-GSCs in response to therapeutic stress including TMZ or ionizing radiation treatment [30, 36]. Another interesting and significant question is whether we should only target GSCs in the treatment of glioma. We suggest this strategy is ineffective, and instead, we should target both GSCs and differentiated glioma cells simultaneously.

Recent studies demonstrated there were many GSCs in the vascular niche, which are important to maintain the GSC phenotype and promote glioma malignant progression [31, 37, 38]. Calabrese and colleagues [39] demonstrated compared with GSC mono-culture, the co-culture of glioma and endothelial cells contributed to GSCs proliferation and malignant tumor formation. VEGF expression in the hypoxic microenvironment is important to vessel formation [37, 40], which is regulated by hypoxia inducible factor-1α (HIF1α). Studies have shown that HIF1α plays an important role in stemness maintenance, angiogenesis and tumorigenesis [2, 9, 13, 37, 41], and we therefore analyzed our results and determined they were in accordance with previous studies. HIF1α was highly expressed in hypoxia and the neurosphere formation rates sharply decreased by approximately 30% when HIF1α was silenced. And then we implanted Gl261 HIF1α-ShRNA cells into mice brain and found the tumor size in this group was much smaller than control with the same tumor cells seeded. We also demonstrated that serum played an important role in the dedifferentiation process because few neurospheres formed without serum. We subsequently detected the VEGF expression in 1% hypoxia and demonstrated that the VEGF expression significantly increased at both mRNA and protein levels. Moreover, the VEGF mRNA and protein levels decreased after HIF1α knockdown. We conclude that HIF1α expresses at first in hypoxia and then promotes the dedifferentiation and vessel formation of glioma cells. These newly formed vessels transport serum and oxygen to the GSCs, which subsequently acquire differentiated features and grow the tumor stepwise. Through the dedifferentiation and differentiation regulated by HIF1α and VEGF, both cell types are dynamic and thus promote glioma growth and increase malignancy. However, another interesting and strange phenomenon why HIF1α expressed at cell cytoplasm not nucleus after hypoxia in this study needs further studies.

Recently, additional studies have demonstrated stemness increased in different microenvironments, and transcription factors such as Sox-2, Oct-4, Klf-4 and Nanog were reprogrammed into both normal tissues including human dermal fibroblasts [42] and Müller glia cells [43], and tumors including lung cancer [44], colon cancer [45] and ovarian cancer [46]. Here, we demonstrated that hypoxia may induce the dedifferentiation of differentiated glioma cells which then acquire the stemness features [9], suggesting that development of therapeutic strategies targeting GSCs should take oxygen tension in full consideration [47].

## MATERIALS AND METHODS

### CD133-CD15-NESTIN- cell isolation and Cell culture

GL261 and U87 cells, as well as primary glioma cells isolated from surgical waste were used as glioma cell lines. Magnetic cell sorting (MACS; Miltenyi Biotech, Bergisch-Gladbach, Germany) was used to isolate CD133^−^CD15^−^NESTIN^−^ cells [34]. In brief, primary glioma, GL261 or U87 cells were incubated in DMEM/F12+10% FBS at 37°C for 3 days, and cell suspensions were prepared following 0.25% trypsin digestion. The cells were subsequently counted and re-suspended in PBS that contained 0.08% EDTA and 0.5% BSA (PBSE; 10^8^ cells/500 μl), followed by incubation for 15 min at 4°C with polyclonal rabbit anti-human CD133^+^ IgGs (Miltenyi Biotech, Germany) or polyclonal rabbit anti-mouse CD133^+^ IgGs (Miltenyi Biotech, Germany). After washing with PBS that contained 1% BSA, the cells were re-suspended in PBSE (10^8^ cells/300 μl), labeled with goat anti-rabbit IgG MicroBeads (Miltenyi Biotech, Germany), incubated for 15 min at 10°C, washed twice with PBSE and re-suspended in 500 μl PBSE. A cell separation column with a flow resistor was subsequently placed in a miniMACS magnet fixed on the MACS multistand and flushed with 500 μl PBSE. The 500 μl cell suspension was poured into the column reservoir. The CD133^+^ cells were retained within the magnet, and unlabeled nonmagnetic CD133^−^ cells passed through the column and were collected. To increase the purity of the CD133^−^ cells, we repeated the previous steps three times. The same methods were used to sort CD15^−^ cells from CD133^−^ cells and NESTIN^−^ cells from CD133^−^CD15^−^ cells. The CD133^−^CD15^−^NESTIN^−^ cells were subsequently cultured in DMEM/F12+10% FBS at 37°C to maintain their differentiated status.

### Clonogenicity and asymmetric division assay

The CD133^−^CD15^−^NESTIN^−^ glioma cells were diluted to 1,500 cells/1 ml DMEM/F12+10% FBS. 1 μl of medium was subsequently transferred to one well of a 96-well plate that had previously been pre-coated with 200 μl serum-free DMEM/F12 culture medium (Figure 1A). Three 96-well plates were incubated at 37°C with 1% O_2_ and 5% CO_2_, and other three plates were incubated with 21% O_2_ and 5% CO_2_. The numbers of surviving cells and newly formed neurospheres were recorded at 0, 3, 7, 14 and 21 days (d). Then we digested neurospheres to single cell suspension with 0.25% trypsin and added 1ml Trypan blue into the cell suspension and observed whether the colour of cell cytoplasm became blue. For asymmetric division, the neurospheres were subsequently transferred to six 24-well plates and half of the samples were cultured with serum-free medium that contained EGF and FGF2, and the other samples were cultured with DMEM/F12+10% FBS. All 24-well plates were incubated at 37°C with 21% O_2_ and 5% CO_2_ and recorded neurospheres morphology at 1, 3 and 5 d.

### Protein detection through immunofluorescence

Newly formed neurospheres and differentiated cells after 48 h of hypoxia were fixed with 4% paraformaldehyde for 10 min at 4°C, washed with PBS and blocked with 10% normal serum for 20 min in PBS that contained 0.5% Triton X-100. The cells were incubated for 24 h at 4°C with primary antibodies against SOX-2 (1:100, MAB2018, R&D Systems, USA), OCT-4 (1:100, MAB1759, R&D Systems, USA), Nanog (1:100, Human: AF1997; Mouse: AF2729, R&D Systems, USA), KLF-4 (1:100, Human: AF3640; Mouse: AF3158, R&D Systems, USA), CD133 (1:150, MBS462020, MyBiosource, USA), CD15 (1:100, MAB2155, R&D Systems, USA), NESTIN (1:100, Human: MAB1259; Mouse: MAB2736, R&D Systems, USA), ABCG2 (1:100, ab130244, Abcam, USA), VEGF (1:100, MAB293, R&D Systems, USA) and HIF1α (1:100, MAB1536, R&D Systems, USA). Neurospheres and cells were washed three times with PBS for 5 min and then incubated at 37°C for 1 h with appropriate fluorophore-labeled secondary antibodies. Images were acquired with a laser scanning confocal microscope (LSM780, ZEISS, Germany).

### Protein detection via Western blot

Cells cultured in hypoxia for 0, 12, 24, 48 and 72 h were collected, subjected to SDS-PAGE and transferred onto nitrocellulose membranes. The membranes were blocked with 5% non-fat milk and incubated with antibodies against SOX-2 (1:1000, MAB2018, R&D Systems, USA), OCT-4 (1:1000, MAB1759, R&D Systems, USA), Nanog (1:1000, Human: AF1997; Mouse: AF2729, R&D Systems, USA), KLF-4 (1:1000, Human: AF3640; Mouse: AF3158, R&D Systems, USA), CD133 (1:1000, MBS462020, MyBiosource, USA), CD15 (1:1000, MAB2155, R&D Systems, USA), NESTIN (1:1000, Human: MAB1259; Mouse: MAB2736, R&D Systems, USA), ABCG2 (1:1000, ab130244, Abcam, USA), VEGF (1:1000, MAB293, R&D Systems, USA) and HIF1α (1:1000, MAB1536, R&D Systems, USA). Enhanced chemiluminescence was conducted for visualization.

### Real-time quantitative polymerase chain reaction

Cells exposed to hypoxia for 0, 3, 6, 9, 12 and 24 h were collected and used to examine the mRNA expression of stem cell markers with RT-PCR. The melting temperature was 94°C, 5 min; the denaturing temperature was 94°C, 30 s; the annealing temperature was 57°C, 30 s; the extending temperature was 72°C, 30 s; in total, 40 cycles were completed. The primer sequences were in Supplementary Table 5.

### Flow cytometry analysis

Flow cytometry (FCM) was used to determine the expression of GSC markers CD133, CD15 and NESTIN in CD133^−^CD15^−^NESTIN^−^ glioma cells exposed to hypoxia for 0, 3, 6, 9, 12 and 15d, HIF1α-ShRNA and HIF1α-vector glioma cells cultured under hypoxia 12d. Cells were collected and incubated with anti-CD133 antibody (Human: Miltenyi Biotech; Mouse: Biolegend, USA), anti-CD15 antibody (FAB2155G-100, R&D Systems, USA) and anti-NESTIN antibody (Human: IC1259P; Mouse: IC2736P, R&D Systems, USA) and analyzed with FCM. For cell cycle and apoptosis analysis, cells cultured in DMEM/F12 with 1%FBS were exposed to normoxia and hypoxia for 0, 1, 3, 5 and 7d; and then collected and detected for FCM analysis.

### Cell growth assay

CCK-8 was used to examine cell growth. Logarithmic phase glioma cells were collected and digested to prepare single cell suspension. The cells were counted and seeded into four 96-well plates (2000 cells/well). Two plates were cultured with normoxia (21% O_2_) and others exposed to hypoxia (1% O_2_). Cell growth was examined on day 1, 3, 5, 7, 9 and 11, respectively. 10μl of CCK-8 solution and 90μl culture medium were mixed and added into each well. The resulting samples were cultured for 2 h respectively under hypoxia or normoxia condition. The absorbance at 450nm was measured with ELISA reader (Varioskan Flash, Thermo Scientific, USA).

### Construction lentiviral luciferase reporters and induced the formation of GL261-Luc

We used the pGL3 reporter plasmid as a template. PCR was conducted using FX-KOD HiFi enzyme constructed with Bgl II-Sal I restriction enzyme cutting sites (as subsequently described). The primers were used to amplify the luciferase gene and for sequencing.

upstream 5′-GAAGATCTCACCATGGAAGACG CCAA-3′

downstream 5′-GCGTCGACTTACACGGCGATC TTTCCGC-3′

The sequenced plasmid was cut by the restriction enzymes. The fragment was sub-cloned into the plenti6-V5-D-TOPO plasmid and packaged into virus. The target Gl261 cells were subsequently infected with virus for 2 days and screened using blasticidin; stably infected Gl261-Luc cells were obtained at approximately 2 weeks.

### Xenograft analysis

We used two different strategies (Figure 5A) to investigate the effect of hypoxia-induced GSCs on tumorigenesis *in vivo*.

Strategy 1: 5 × 10^3^ differentiated CD133^−^CD15^−^NESTIN^−^ GL261-Luc cells were seeded into each well of 6-well plates that had previously been pre-coated with 2 ml serum-free DMEM/F12 culture medium and cultured under hypoxia conditions 14d; then collected and digested neurospheres with 0.25% trypsin to single cell suspension. Counted and injected 10^4^ cells into the right frontal lobe of 6-week-old C57 female mice (*n* = 25) that were raised under normoxia. Fifteen mice were used to examine the survival rate, and the remaining mice were used to investigate tumor growth by the bioluminescence of cancer cells at 5, 10, 15, 20 and 25 d. To determine the amount of bioluminescence, 200 μl (15 mg/1 ml) of luciferins were administered via peritoneal injection to mice under anesthesia, and the bioluminescence was detected using the NightOWL Macro Imaging system (LB983 NC320, Berthold Technologies, Germany). For control group, cells cultured under normoxia (21% O_2_) were injected into the same brain region of the mice (*n* = 21) and examined using identical procedures.

Strategy 2: We injected 3 × 10^4^ CD133^−^CD15^−^NESTIN^−^ GL261-Luc cells exposed to normoxia into age-matched C57 mouse brains. The mice were raised under normoxia (21% O_2_; *n* = 22) or hypoxia (10% O_2_; *n* = 21). On 5, 10, 15, 20 and 25 d, bioluminescence was measured as previously described.

All animals were raised for 30 days, and the 30 day survival rate was assessed. To rule out the influence of other factors on mice, 30 additional mice were raised in 21% O_2_ or 10% O_2_ without tumor cells. Immunohistochemistry, RT-PCR and Western blot were used to examine the stem cell markers and HIF1α expression.

### Influence of serum on clone formation

Cell suspensions were diluted to 1,500 cells/1 ml DMEM/F12+10% FBS or 1,500 cells/1 ml DMEM/F12 (Figure 8A). Single glioma cell sphere formation assay were defined as previously described. All 96-well plates were incubated under 1% O_2_; the numbers of surviving cells and newly formed neurospheres were recorded at 0, 3, 7, 14 and 21 d. Cell death rate was examined at 1, 3, 5 and 7 d.

### HIF1α interfere assays

The HIF1α-ShRNA lentivirus was purchased from Cyagen, Guangzhou, China. CD133^−^CD15^−^NESTIN^−^ glioma cells were counted, and 10^5^ CD133^−^CD15^−^NESTIN^−^ cells were seeded into 6-well plates. HIF1α-ShRNA-lentivirus (MOI=1) was added to 6-well plates following cell attachment for 24 h. 1 μg/ml of puromycin was subsequently added to the 6-well plates following lentiviral infection for 48 h. This process lasted one week. The cells were then collected, and HIF1α expression was detected via Western blot. Successful HIF1α-interference cells were cultured in DMEM/F12+10% FBS to maintain growth. Digoxin (TOCRIS, 4583) was dissolved at 100 mM in DMSO, and the effective concentration was 100 nm/L [24, 48]. Successful HIF1α-interference cells were detected via Western blot. To determine the influence of low HIF1α expression on neurosphere formation in hypoxia, we performed single glioma cell sphere formation assays as previously described. Moreover, we also determined VEGF expression of HIF1α interference cells following 1% hypoxia using RT-PCR and Western blot. For *in vivo* study, we implanted 3 × 10^4^ HIF1α-ShRNA CD133^−^CD15^−^NESTIN^−^ GL261 cells (Group1, *n* = 30), HIF1α-Vector CD133^−^CD15^−^NESTIN^−^ GL261 cells (Group 2, *n* = 30) and CD133^−^CD15^−^NESTIN^−^ GL261 cells (Group 3, *n* = 30) respectively into C57 mice brain. Fifteen mice of each group were used to examine survival rate, and the remaining mice were used to investigate tumor growth by the bioluminescence of cancer cells at 20 d as identical procedures above.

### Statistical analysis

SPSS 19.0 was used for statistical analysis. Data were expressed as the means ± standard deviations (SDs). Differences were evaluated with *T* test and one-way analysis of variance (ANOVA) when necessary. A log-rank test was used to assess the survival rate and *P <* 0.05 was considered statistically significant.

## SUPPLEMENTARY MATERIALS FIGURES AND TABLES



## References

[1] Mansour J, Fields B, Macomson S, Rixe O (2014). Significant anti-tumor effect of bevacizumab in treatment of pineal gland glioblastoma multiforme. Targeted oncology.

[2] Li XT, Tang W, Jiang Y, Wang XM, Wang YH, Cheng L, Meng XS (2016). Multifunctional targeting vinorelbine plus tetrandrine liposomes for treating brain glioma along with eliminating glioma stem cells. Oncotarget.

[3] Medema JP (2013). Cancer stem cells: the challenges ahead. Nat Cell Biol.

[4] Singh SK, Clarke ID, Terasaki M, Bonn VE, Hawkins C, Squire J, Dirks PB (2003). Identification of a cancer stem cell in human brain tumors. Cancer Res.

[5] Singh SK, Hawkins C, Clarke ID, Squire JA, Bayani J, Hide T, Henkelman RM, Cusimano MD, Dirks PB (2004). Identification of human brain tumour initiating cells. Nature.

[6] Sgubin D, Wakimoto H, Kanai R, Rabkin SD, Martuza RL (2012). Oncolytic herpes simplex virus counteracts the hypoxia-induced modulation of glioblastoma stem-like cells. Stem cells translational medicine.

[7] Denko NC (2008). Hypoxia, HIF1 and glucose metabolism in the solid tumour. Nat Rev Cancer.

[8] Pistollato F, Abbadi S, Rampazzo E, Persano L, Della Puppa A, Frasson C, Sarto E, Scienza R, D’Avella D, Basso G (2010). Intratumoral hypoxic gradient drives stem cells distribution and MGMT expression in glioblastoma. Stem cells (Dayton Ohio).

[9] Li P, Zhou C, Xu L, Xiao H (2013). Hypoxia enhances stemness of cancer stem cells in glioblastoma: an *in vitro* study. International journal of medical sciences.

[10] Jögi A, Øra I, Nilsson H, Lindeheim A, Makino Y, Poellinger L, Axelson H, Påhlman S (2002). Hypoxia alters gene expression in human neuroblastoma cells toward an immature and neural crest-like phenotype. Proc Natl Acad Sci USA.

[11] Molina ES, Pillat MM, Moura-Neto V, Lah TT, Ulrich H (2014). Glioblastoma stem-like cells: approaches for isolation and characterization. Journal of Cancer Stem Cell Research.

[12] Nissou MF, El Atifi M, Guttin A, Godfraind C, Salon C, Garcion E, van der Sanden B, Issartel JP, Berger F, Wion D (2013). Hypoxia-induced expression of VE-cadherin and filamin B in glioma cell cultures and pseudopalisade structures. J Neurooncol.

[13] Qiang L, Wu T, Zhang HW, Lu N, Hu R, Wang YJ, Zhao L, Chen FH, Wang XT, You QD, Guo QL (2012). HIF-1alpha is critical for hypoxia-mediated maintenance of glioblastoma stem cells by activating Notch signaling pathway. Cell death and differentiation.

[14] Bao S, Wu Q, McLendon RE, Hao Y, Shi Q, Hjelmeland AB, Dewhirst MW, Bigner DD, Rich JN (2006). Glioma stem cells promote radioresistance by preferential activation of the DNA damage response. Nature.

[15] Piccirillo SG, Spiteri I, Sottoriva A, Touloumis A, Ber S, Price SJ, Heywood R, Francis NJ, Howarth KD, Collins VP, Venkitaraman AR, Curtis C, Marioni JC (2015). Contributions to drug resistance in glioblastoma derived from malignant cells in the sub-ependymal zone. Cancer Res.

[16] Bao S, Wu Q, Li Z, Sathornsumetee S, Wang H, McLendon RE, Hjelmeland AB, Rich JN (2008). Targeting cancer stem cells through L1CAM suppresses glioma growth. Cancer Res.

[17] Bleau AM, Hambardzumyan D, Ozawa T, Fomchenko EI, Huse JT, Brennan CW, Holland EC (2009). PTEN/PI3K/Akt pathway regulates the side population phenotype and ABCG2 activity in glioma tumor stem-like cells. Cell stem cell.

[18] Ben-Porath I, Thomson MW, Carey VJ, Ge R, Bell GW, Regev A, Weinberg RA (2008). An embryonic stem cell-like gene expression signature in poorly differentiated aggressive human tumors. Nat Genet.

[19] Fornara O, Bartek J, Rahbar A, Odeberg J, Khan Z, Peredo I, Hamerlik P, Bartek J, Stragliotto G, Landázuri N, Söderberg-Nauclér C (2015). Cytomegalovirus infection induces a stem cell phenotype in human primary glioblastoma cells: prognostic significance and biological impact. Cell Death Differ.

[20] Parajuli P, Anand R, Mandalaparty C, Suryadevara R, Sriranga PU, Michelhaugh SK, Cazacu S, Finniss S, Thakur A, Lum LG, Schalk D, Brodie C, Mittal S (2016). Preferential expression of functional IL-17R in glioma stem cells: potential role in self-renewal. Oncotarget.

[21] Wang Z, Yang J, Xu G, Wang W, Liu C, Yang H, Yu Z, Lei Q, Xiao L, Xiong J, Zeng L, Xiang J, Ma J (2015). Targeting miR-381-NEFL axis sensitizes glioblastoma cells to temozolomide by regulating stemness factors and multidrug resistance factors. Oncotarget.

[22] Qiang L, Yang Y, Ma YJ, Chen FH, Zhang LB, Liu W, Qi Q, Lu N, Tao L, Wang XT, You QD, Guo QL (2009). Isolation and characterization of cancer stem like cells in human glioblastoma cell lines. Cancer letters.

[23] Koh MY, Lemos R, Liu X, Powis G (2011). The hypoxia-associated factor switches cells from HIF-1α- to HIF-2α-dependent signaling promoting stem cell characteristics, aggressive tumor growth and invasion. Cancer Res.

[24] Bar EE, Lin A, Mahairaki V, Matsui W, Eberhart CG (2010). Hypoxia increases the expression of stem-cell markers and promotes clonogenicity in glioblastoma neurospheres. The American journal of pathology.

[25] Kumar H, Choi DK (2015). Hypoxia Inducible Factor Pathway and Physiological Adaptation: A Cell Survival Pathway?. Mediators of Inflammation.

[26] Blazek ER, Foutch JL, Maki G (2007). Daoy medulloblastoma cells that express CD133 are radioresistant relative to CD133- cells, and the CD133+ sector is enlarged by hypoxia. International journal of radiation oncology, biology, physics.

[27] Mathieu J, Zhang Z, Zhou W, Wang AJ, Heddleston JM, Pinna CM, Hubaud A, Stadler B, Choi M, Bar M, Tewari M, Liu A, Vessella R (2011). HIF induces human embryonic stem cell markers in cancer cells. Cancer research.

[28] Chen J, Li Y, Yu TS, McKay RM, Burns DK, Kernie SG, Parada LF (2012). A restricted cell population propagates glioblastoma growth after chemotherapy. Nature.

[29] Wang J, Sakariassen P, Tsinkalovsky O, Immervoll H, Bøe SO, Svendsen A, Prestegarden L, Røsland G, Thorsen F, Stuhr L, Molven A, Bjerkvig R, Enger P (2008). CD133 negative glioma cells form tumors in nude rats and give rise to CD133 positive cells. Int J Cancer.

[30] Auffinger B, Tobias AL, Han Y, Lee G, Guo D, Dey M, Lesniak MS, Ahmed AU (2014). Conversion of differentiated cancer cells into cancer stem-like cells in a glioblastoma model after primary chemotherapy. Cell Death Differ.

[31] Fessler E, Borovski T, Medema JP (2015). Endothelial cells induce cancer stem cell features in differentiated glioblastoma cells via bFGF. Mol Cancer.

[32] Matsuda Y, Ishiwata T, Yoshimura H, Hagio M, Arai T (2015). Inhibition of nestin suppresses stem cell phenotype of glioblastomas through the alteration of post-translational modification of heat shock protein HSPA8/HSC71. Cancer Lett.

[33] Gao X, McDonald JT, Hlatky L, Enderling H (2013). Acute and fractionated irradiation differentially modulate glioma stem cell division kinetics. Cancer research.

[34] Beier D, Hau P, Proescholdt M, Lohmeier A, Wischhusen J, Oefner PJ, Aigner L, Brawanski A, Bogdahn U, Beier CP (2007). CD133(+) and CD133(−) glioblastoma-derived cancer stem cells show differential growth characteristics and molecular profiles. Cancer research.

[35] Li Y, Li A, Glas M, Lal B, Ying M, Sang Y, Xia S, Trageser D, Guerrero-Cázares H, Eberhart CG, Quiñones-Hinojosa A, Scheffler B, Laterra J (2011). c-Met signaling induces a reprogramming network and supports the glioblastoma stem-like phenotype. Proc Natl Acad Sci USA.

[36] Dahan P, Martinez Gala J, Delmas C, Monferran S, Malric L, Zentkowski D, Lubrano V, Toulas C, Cohen-Jonathan Moyal E, Lemarie A (2014). Ionizing radiations sustain glioblastoma cell dedifferentiation to a stem-like phenotype through survivin: possible involvement in radioresistance. Cell Death & Disease.

[37] Lathia JD, Heddleston JM, Venere M, Rich JN (2011). Deadly teamwork: neural cancer stem cells and the tumor microenvironment. Cell stem cell.

[38] Infanger DW, Cho Y, Lopez BS, Mohanan S, Liu SC, Gursel D, Boockvar JA, Fischbach C (2013). Glioblastoma stem cells are regulated by interleukin-8 signaling in a tumoral perivascular niche. Cancer Res.

[39] Calabrese C, Poppleton H, Kocak M, Hogg TL, Fuller C, Hamner B, Oh EY, Gaber MW, Finklestein D, Allen M, Frank A, Bayazitov IT, Zakharenko SS (2007). A perivascular niche for brain tumor stem cells. Cancer Cell.

[40] Hjelmeland AB, Wu Q, Heddleston JM, Choudhary GS, MacSwords J, Lathia JD, McLendon R, Lindner D, Sloan A, Rich JN (2011). Acidic stress promotes a glioma stem cell phenotype. Cell Death Differ.

[41] Soeda A, Park M, Lee D, Mintz A, Androutsellis-Theotokis A, McKay RD, Engh J, Iwama T, Kunisada T, Kassam AB, Pollack IF, Park DM (2009). Hypoxia promotes expansion of the CD133-positive glioma stem cells through activation of HIF-1alpha. Oncogene.

[42] Takahashi K, Tanabe K, Ohnuki M, Narita M, Ichisaka T, Tomoda K, Yamanaka S (2007). Induction of pluripotent stem cells from adult human fibroblasts by defined factors. Cell.

[43] Wan J, Ramachandran R, Goldman D (2012). HB-EGF is necessary and sufficient for Müller glia dedifferentiation and retina regeneration. Dev Cell.

[44] Chiou SH, Wang ML, Chou YT, Chen CJ, Hong CF, Hsieh WJ, Chang HT, Chen YS, Lin TW, Hsu HS, Wu CW (2010). Coexpression of Oct4 and Nanog enhances malignancy in lung adenocarcinoma by inducing cancer stem cell-like properties and epithelial-mesenchymal transdifferentiation. Cancer Res.

[45] King CE, Cuatrecasas M, Castells A, Sepulveda AR, Lee JS, Rustgi AK (2011). LIN28B promotes colon cancer progression and metastasis. Cancer Res.

[46] Choi YJ, Ingram PN, Yang K, Coffman L, Iyengar M, Bai S, Thomas DG, Yoon E, Buckanovich RJ (2015). Identifying an ovarian cancer cell hierarchy regulated by bone morphogenetic protein 2. Proc Natl Acad Sci USA.

[47] Albert I, Hefti M, Luginbuehl V (2014). Physiological oxygen concentration alters glioma cell malignancy and responsiveness to photodynamic therapy *in vitro*. Neurological research.

[48] Zhang H, Qian DZ, Tan YS, Lee K, Gao P, Ren YR, Rey S, Hammers H, Chang D, Pili R, Dang CV, Liu JO, Semenza GL (2008). Digoxin and other cardiac glycosides inhibit HIF-1alpha synthesis and block tumor growth. Proceedings of the National Academy of Sciences of the United States of America.

