# Supplementary material for: HIF1α regulates single differentiated glioma cell dedifferentiation to stem-like cell phenotypes with high tumorigenic potential under hypoxia
Source: Oncotarget. 2017 Mar 3;8(17):28074–92. doi: 10.18632/oncotarget.15888 (PMC5438632; doi:10.18632/oncotarget.15888)
Supplement: Supplementary file 1 [file oncotarget-08-28074-s001.pdf]

HIF1α regulates single differentiated glioma cell dedifferentiation to stem-like cell phenotypes with high tumorigenic potential under hypoxia

Supplementary Materials

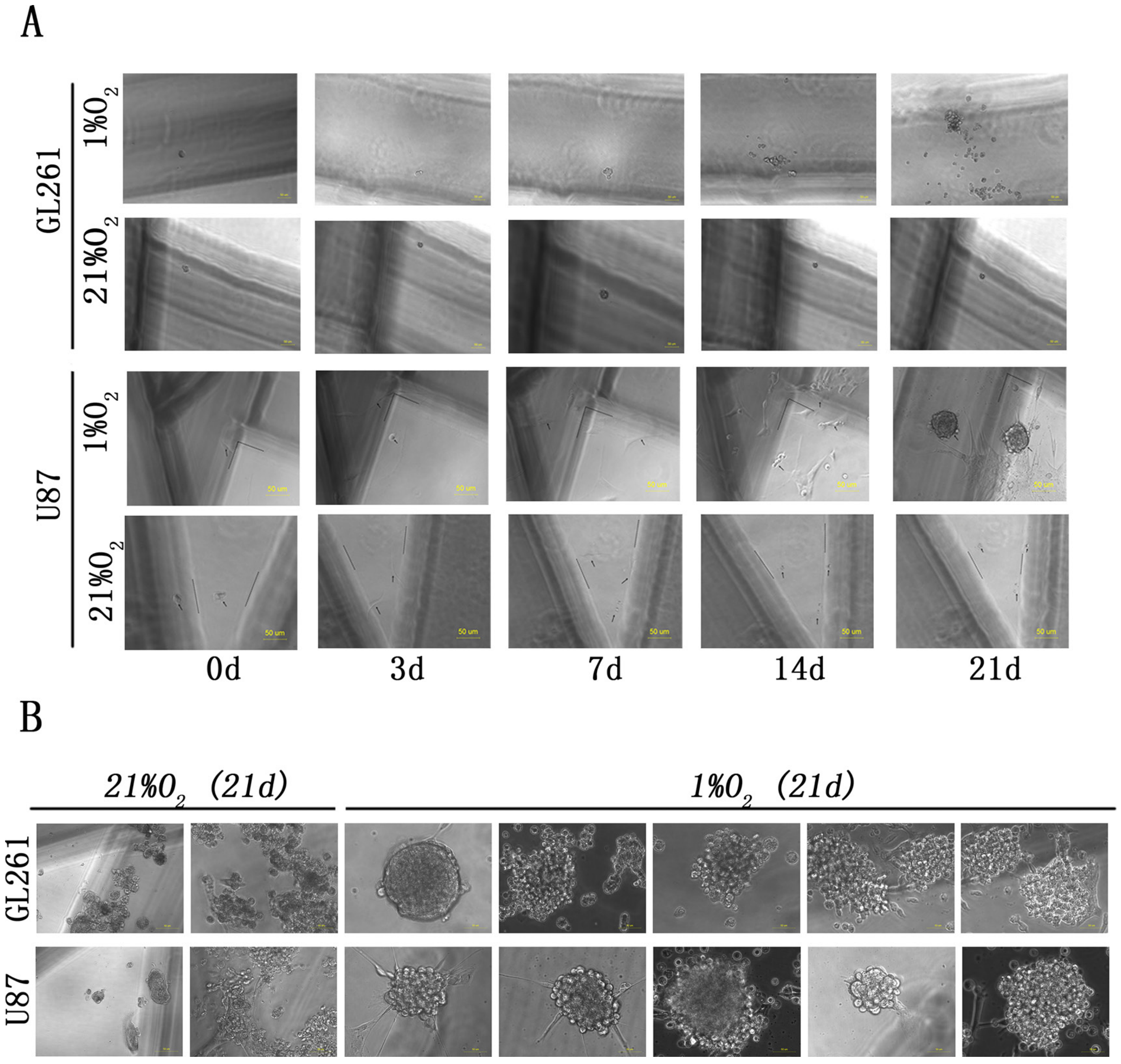

Supplementary Figure 1: Dedifferentiation can be induced under hypoxia conditions. (**A**) We recorded cell status under hypoxia and normoxia on day 0, 3, 7, 14 and 21 and found hypoxia (1%O_2) induced the formation of neurospheres by Single $CD133^-CD15^-NESTIN^-$ GL261 or U87 cell, but no neurosphere formed under normoxia (21%O_2) (**B**) Representative pictures of irregular, non-adherent and loose aggregates under normoxia and representative pictures of induced neurospheres under hypoxia.

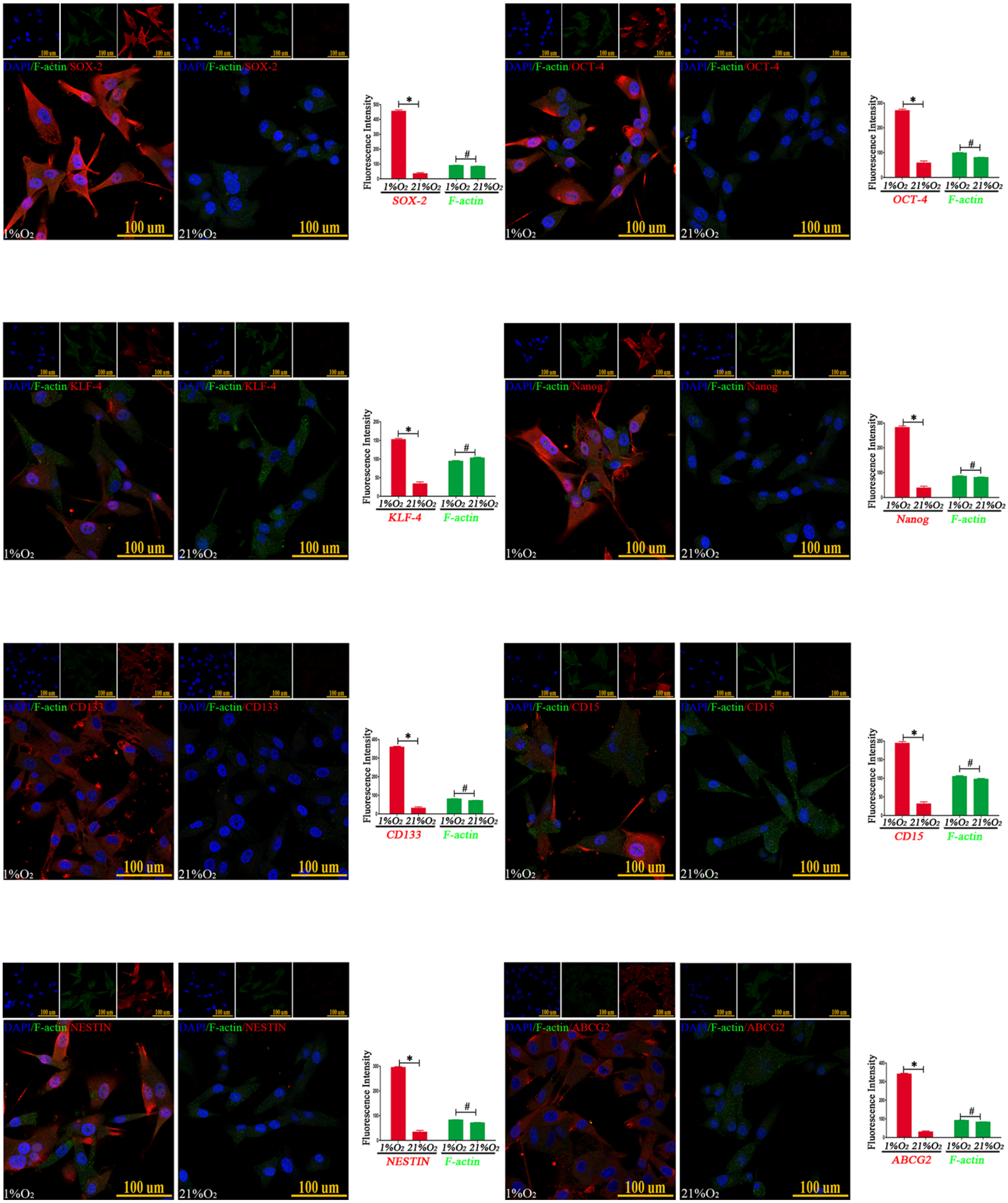

Supplementary Figure 2: Hypoxia improved the expression of stem cell markers in the detection of immunofluorescence. To improve the accuracy and set the detection in the same background in immunofluorescence, we did double immunofluorescent labeling of f-actin and stem cell markers for U87 $CD133^{-}CD15^{-}NESTIN^{-}$ cells cultured in hypoxia and normoxia and the results showed there were no difference for the expression of f-actin between hypoxia and normoxia group ($^{\#}P > 0.05$, Paired-samples *T* Test); however, significant higher expression were demonstrated for SOX-2, OCT-4, KLF-4, Nanog, CD133, CD15, NESTIN and ABCG2 in the cells under hypoxia compared with the expression of stem cell markers of normoxia treated cells ($^{*}P < 0.05$, Paired-samples *T* Test).

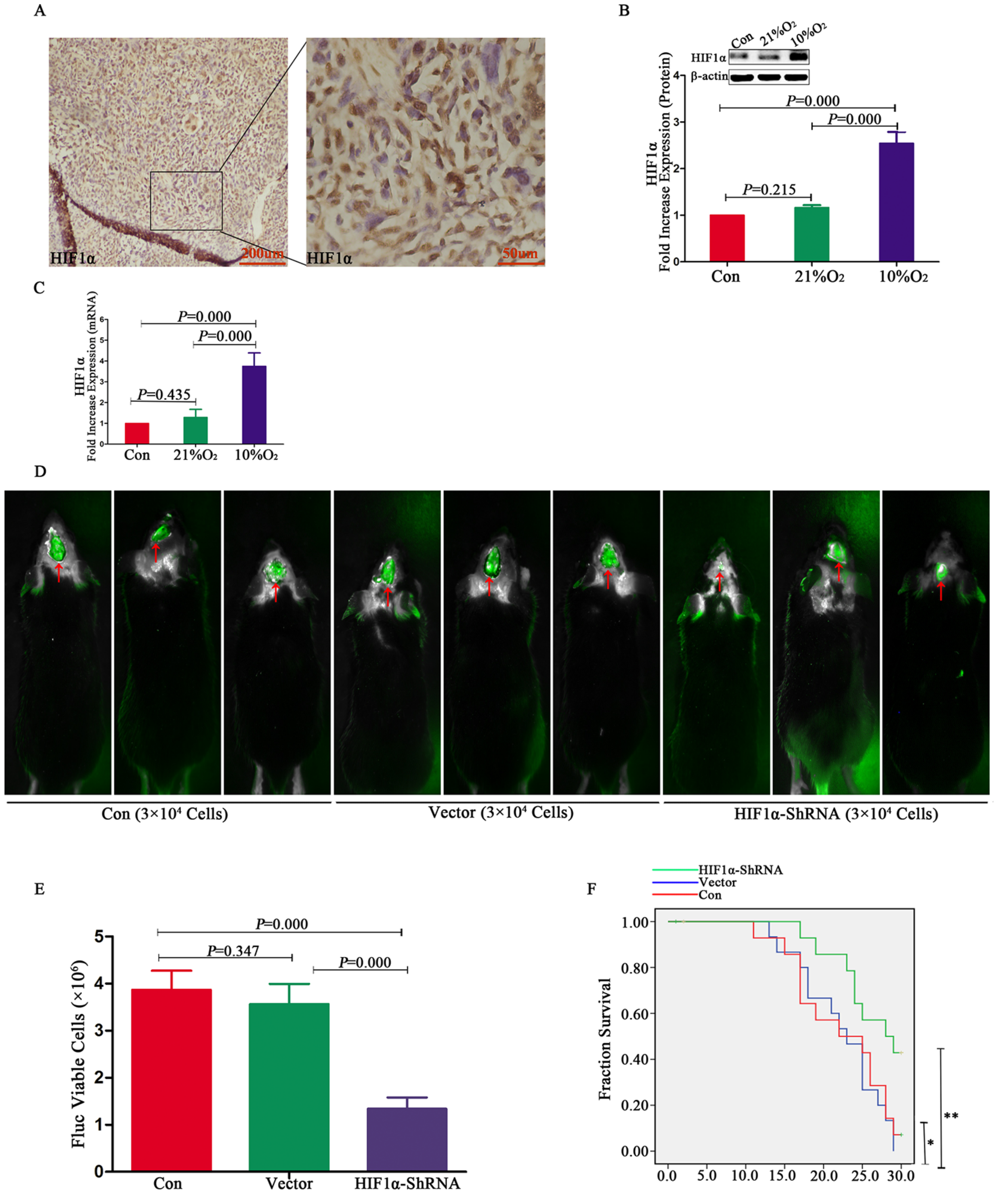

Supplementary Figure 3: The influence of HIF1α on tumor formation. (**A**) Immunohistochemical staining demonstrated there was HIF1α expression in tumor sample collected from mice fed in 10%O_2. (**B**) Western-blot showed HIF1α expression of tumor sample collected from mice fed in 10%O_2 was about three times higher than control including normal brain tissues and tumor sample obtained from normoxia (21%O_2) raised mice. (**C**) RT-PCR showed there was higher expression of HIF1α in the tumor sample collected from mice fed in 10%O_2, and no differences was detected between normal brain tissues and tumor sample obtained from normoxia (21%O_2) raised mice. (**D–E**) GL261-luc HIF1α-ShRNA cells seeded group raised in 10%O_2 showed a smaller size of tumor formation than control and the quantitative value of bioluminescence was significantly decreased ($*P < 0.001$, Paired-samples *T* Test). (**F**) The survival rate of GL261 HIF1α-ShRNA cells seeded group was higher than control ($*P > 0.05$, $**P < 0.05$, Log-rank Test)

Supplementary Table 1: Single CD133-CD15-NESTIN- cell seeded and neurospheres formed under normoxia or hypoxia

A

Normoxia (21% O_2)

	trials	0d seeding	3d Survived cell	3d aggregates	7d aggregates	14d aggregates	21d aggregates	d21 aggregates /d3 Survived
**GL261 (3 plates/ trial)**	1	240	142	0	2	5	9	6.34
	2	256	161	0	0	4	7	4.35
	3	252	157	0	0	2	7	4.46
	mean	249.33	153.33	0.00	**0.67**	**3.67**	**7.67**	**5.05**
	SE	8.33	10.02	0.00	1.15	1.53	1.15	1.12
U87 (3 plates/ trial)	1	230	137	0	0	0	0	0.00
	2	261	155	0	0	1	2	1.29
	3	238	152	0	0	1	4	2.63
	mean	243.00	148	0.00	**0.00**	**0.67**	**2.00**	**1.31**
	SE	16.09	9.64	0.00	0.00	0.58	2.00	1.32

B

Hypoxia (1% O_2)

	trials	0d seeding	3d Survived cell	3d Spheres	7d Spheres	14d Spheres	21d Spheres	d21 Spheres /d3 Survived
GL261 (3 plates/ trial)	1	252	149	1	41	99	145	97.32
	2	272	165	2	57	102	165	100.00
	3	241	152	2	39	92	135	88.80
	mean	255.00	155.33	1.67	**45.67**	**97.67**	**148.33**	**95.38**
	SE	15.72	8.50	0.58	9.87	5.13	15.28	5.83
U87 (3 plates/ trial)	1	261	144	0	52	106	142	98.61
	2	254	156	1	49	99	156	100.00
	3	254	154	3	43	92	152	98.70
	mean	256. 33	151.33	1.33	**48.00**	**99.00**	**150.00**	**99.10**
	SE	4.04	6.43	1.53	4.58	7.00	7.21	0.78

Under hypoxia, >60% CD133$^-$CD15$^-$NESTIN$^-$ cells survived after exposure 3d for both GL261 and U87 glioma cells; and most surviving cells (95.38%±5.83 from GL261 and 99.10%±0.78 from U87) formed neurospheres at 21 d. However, under normoxia, only 5.05%±1.12 from GL261 and 1.31%±1.32 from U87 cells formed sparse, irregular and non-adherent aggregates.

Supplementary Table 2: Numerical value of the Fluc-Viable glioma cells in groups

Tumor Size / Time (d) / Group	5d	10d	15d	20d	25d
1	8.280×10^4<br>2.819×10^4<br>2.101×10^4	31.840×10^4<br>8.718×10^4<br>2.209×10^4	4.211×10^4<br>6.185×10^4<br>29.810×10^4	523.100×10^4<br>294.200×10^4<br>156.100×10^4	399.000×10^4<br>334.200×10^4<br>62.770×10^4
2	1.814×10^4<br>1.636×10^4<br>1.613×10^4	3.272×10^4<br>2.761×10^4<br>4.046×10^4	0.775×10^4<br>3.756×10^4<br>4.593×10^4	2.940×10^4<br>1.957×10^4<br>2.844×10^4	1.086×10^4<br>0.976×10^4<br>1.246×10^4
3	2.624×10^4<br>2.678×10^4<br>4.271×10^4	8.684×10^4<br>14.990×10^4<br>14.230×10^4	47.140×10^4<br>1.466×10^4<br>31.600×10^4	87.690×10^4<br>12.810×10^4<br>3.273×10^4	0.416×10^4<br>0.551×10^4<br>2.795×10^4
4	3.946×10^4<br>4.757×10^4<br>6.010×10^4	19.680×10^4<br>20.880×10^4<br>25.540×10^4	99.870×10^4<br>30.450×10^4<br>90.930×10^4	126.000×10^4<br>19.990×10^4<br>536.500×10^4	545.500×10^4<br>258.100×10^4<br>11.390×10^4

Group 1: C57 Mouse (21%O_2); GL261 Cell (1%O_2)
Group 2: C57 Mouse (21%O_2); GL261 Cell (21%O_2) } STRATEGY 1

Group 3: C57 Mouse (21%O_2); GL261 Cell (21%O_2)
Group 4: C57 Mouse (10%O_2); GL261 Cell (21%O_2) } STRATEGY 2

Two different strategies were used to investigate the effect of hypoxia-induced GSCs on tumorigenesis in vivo. STRATEGY 1 (Group1-Group2): 14d hypoxia (1%)-induced neurospheres–derived GL261 cells (Group1 10^4) or normoxia (21%)-derived GL261 $CD133^-CD15^-NESTIN^-$ cells (Group2 10^4) were injected into the brains of adult female C57 mice and these mice were raised under normoxia (21% O_2). There were no significant differences on tumor volume between the two groups on day 5; but after 25d, tumors were detected in group 1 with magnitude orders more than 10^6. For group 2 the orders of magnitude were around 10^4. STRATEGY 2 (Group3-Group4): the mice in group 3 (3×10^4) underwent similar changes as group 2 in strategy 1, and group 4 was injected with differentiated $CD133^-CD15^-NESTIN^-$ Gl261 cells (3×10^4) but raised under 10% O_2 at 25 d, tumors were detected in group 4 with magnitude orders greater than 10^6. For group 3 the orders of magnitude were less than 10^4 and only reached to 4,161.

Supplementary Table 3: Single CD133⁻CD15⁻NESTIN⁻ cell seeded and neurospheres formed under hypoxia contained FBS or not

A

1%O_2(No FBS)

	trials	0d seeding	3d Survived cell	3d aggregates	7d aggregates	14d aggregates	21d aggregates	d21 aggregates /d3 Survived
GL261	1	263	102	0	1	4	9	8.82
	2	251	95	0	0	2	5	5.26
	3	257	92	0	0	0	1	1.09
	mean	257	96.00	0	**0.33**	**2.00**	**5.00**	**5.06**
	SE	6	5.13	0	0.58	2	4	3.87
U87	1	272	113	1	1	4	7	6.19
	2	251	105	0	2	3	5	4.76
	3	263	103	0	0	3	5	4.85
	mean	262.00	107	0.33	**1.00**	**3.33**	**5.67**	**5.27**
	SE	10.54	5.29	0.58	1.00	0.58	1.15	0.80

B

1%O_2(Contained FBS)

	trials	0d seeding	3d Survived cell	3d Spheres	7d Spheres	14d Spheres	21d Spheres	d21 Spheres /d3 Survived
GL261	1	252	149	1	41	99	145	97.32
	2	272	165	2	57	102	165	100.00
	3	241	152	2	39	92	135	88.80
	mean	255.00	155.33	1.67	**45.67**	**97.67**	**148.33**	**95.38**
	SE	15.72	8.50	0.58	9.87	5.13	15.28	5.83
U87	1	261	144	0	52	106	142	98.61
	2	254	156	1	49	99	156	100.00
	3	254	154	3	43	92	152	98.70
	mean	256. 33	151.33	1.33	**48.00**	**99.00**	**150.00**	**99.10**
	SE	4.04	6.43	1.53	4.58	7.00	7.21	0.78

The clone formation rates (spheres/d3 viable cells) were 5.06% ± 3.87 from GL261 and 5.27% ± 0.80 from U87 cells suspended in medium without FBS following hypoxia treatment 21d. However, the clone formation rates (spheres/d3 viable cells) of GL261 and U87 suspended with DMEM/F12+10% FBS were more than 95% after hypoxia treatment 21 d.

Supplementary Table 4: Single U87 CD133-CD15-NESTIN-HIF1α-ShRNA cell seeded and spheres formed under hypoxia

Hypoxia (1%O_2)

I	trials	0d seeding	3d Survived cell	3d Spheres	7d Spheres	14d Spheres	21d Spheres	d21 Spheres /d3 Survived
I	1	243	134	6	52	92	132	98.51
	2	234	144	5	61	97	144	100.00
	3	219	133	9	49	85	129	96.99
	mean	232.00	137.00	6.67	**54.00**	**91.33**	**135**	**98.5**
	SE	12.12	6.08	2.08	6.24	6.03	7.94	1.51
II	1	257	155	9	61	104	148	95.48
	2	229	127	6	55	89	125	98.43
	3	237	139	11	45	82	137	98.56
	mean	241.00	140.00	8.67	**53.67**	**91.67**	**136.67**	**97.49**
	SE	14.42	14.05	2.52	8.08	11.24	11.50	1.74

	trials	0d seeding	3d Survived cell	3d Spheres	7d Spheres	14d Spheres	21d Spheres	d21 Spheres /d3 Survived
III	1	253	141	1	23	53	86	60.99
	2	247	136	3	19	58	92	67.65
	3	257	129	7	12	61	80	62.02
	mean	252.00	135.00	3.67	**18.00**	**57.33**	**86.00**	**63.55**
	SE	5.03	6.03	3.06	5.57	4.04	6.00	3.59
IV	1	272	137	0	0	1	2	1.46
	2	261	119	0	1	1	2	1.68
	3	243	112	0	2	2	3	2.68
	mean	259.00	123.00	0.00	**1.00**	**1.33**	**2.33**	**1.94**
	SE	14.64	12.90	0.00	1.00	0.58	0.58	0.65

I: U87 Not-Targeted HIF1α (1%O_2);
II: U87 HIF1α-Vector 3(1%O_2);
III: U87 HIF1α-ShRNA 3(1%O_2);
IV: Digoxin (1%O_2)

The neurosphere formation rate of the HIF1α-ShRNA- or digoxin-treated U87 cells was substantially lower than that of the controls. The numerical value was 63.55 ± 3.59% from the single $CD133^-CD15^-NESTIN^-$ HIF1α-ShRNA U87 cells and 1.94 ± 0.65% from digoxin treatment U87 cells.

Supplementary Table 5: the primer sequences of stem cell markers

	upstream (5′→3′)	downstream (5′→3′)
Human SOX-2	GGAGGGGTGCAAAAGAGGAGAG	TCCCCCAAAAAGAAGTCCAGG
Human OCT-4	CCCGCCGTATGAGTTCTGTGG	CCGGGTTTTGCTCCAGCTTCTC
Human KLF-4	GGCTGCGGCAAAACCTACAC	CGGGCGAATTTCCATCCAC
Human Nanog	CCGCGCCCTGCCTAGAAAAGAC	AGCCTCCCAATCCCAAACAATACG
Human CD133	GCCCCCAGGAAATTTGAGGAAC	GCTTTGGTATAGAGTGCTCAGTGATTG
Human CD15	TGGGCAGGCTGGTCTTGAACT	CACGGCGGCTCACACCTGTA
Human NESTIN	GCCCCTGGTGGAAGATGATG	GCCCTGAACCCTCTTTGCCTC
Human ABCG2	TAGTGAGGAAAGTTCCTGTC	AGCTCAGTTAACTCCTGTAAG
Human HIF1α	CCCTCACCCACAAAAATTAC	GGGACTATTAGGCTCAGGTGAAC
Human VEGF	TTCGGGAACCAGATCTCTCACC	CGGACCCAAAGTGCTCTGC
Mouse SOX-2	GGGGGCAGCGGCGTAAGATG	CCCGCTCGCCATGCTGTTC
Mouse OCT-4	GCCCGGAAGAGAAAGCGAAC	GGGGCAGAGGAAAGGATACAG
Mouse KLF-4	CCGGCCCAACACACACGACTTC	GAACCCGGTGGCATGAGCTCTTG
Mouse Nanog	GCCCAGCTGTGTGCACTCAAGG	GGCTTCCAGATGCGTTCACCAGATA
Mouse CD133	CCGCGATGGACTCTGCTGTTAATG	GGGCACAGTCTCAACATCGTCGTATAC
Mouse CD15	ATCGGGCTGCTGCACACTG	AGCGGAAGTAGCGGCGATAGAC
Mouse NESTIN	GCCCAAGCAGGTGAACAAGACT	CAGCCCTTGCATTCCAGAGTCT
Mouse ABCG2	AGAAACTCTTCATACATGAGTACA	AAGTGTTGCTACAGACACCACAC
Mouse HIF1α	AGCCTTAACCTGTCTGCCACTTTG	GGGCACAGTCTCAACATCGTCGTATAC
Mouse VEGF	CCGGTTTAAATCCTGGAGCGTTC	CACCGCCTTGGCTTGTCACA
β-actin	ACCCGCCGCCAGCTCACC	GGGGGGCACGAAGGCTCATC
